# Association Between Vitamin D Supplementation During Pregnancy and Offspring Growth, Morbidity, and Mortality: A Systematic Review and Meta-analysis

**DOI:** 10.1001/jamapediatrics.2018.0302

**Published:** 2018-05-29

**Authors:** Wei Guang Bi, Anne Monique Nuyt, Hope Weiler, Line Leduc, Christina Santamaria, Shu Qin Wei

**Affiliations:** 1 Centre Hospitalier Universitaire Saint-Justine Research Center, University of Montréal, Montréal, Quebec, Canada; 2 Department of Obstetrics and Gynecology, University of Montréal, Montréal, Quebec, Canada; 3 Department of Pediatrics; Faculty of Medicine, University of Montréal, Montréal, Quebec, Canada; 4 School of Human Nutrition, McGill University, Montréal, Quebec, Canada

## Abstract

**Importance:**

Whether vitamin D supplementation during pregnancy is beneficial and safe for offspring is unclear.

**Objective:**

To systematically review studies of the effects of vitamin D supplementation during pregnancy on offspring growth, morbidity, and mortality.

**Data Sources:**

Searches of Medline, Embase, and the Cochrane Database of Systematic Reviews were conducted up to October 31, 2017. Key search terms were *vitamin D*, *pregnancy*, *randomized controlled trials*, and *offspring outcomes*.

**Study Selection:**

Randomized clinical trials of vitamin D supplementation during pregnancy and offspring outcomes.

**Data Extraction and Synthesis:**

Two authors independently extracted data, and the quality of the studies was assessed. Summary risk ratio (RR), risk difference (RD) or mean difference (MD), and 95% CI were calculated using fixed-effects or random-effects meta-analysis.

**Main Outcomes and Measures:**

Main outcomes were fetal or neonatal mortality, small for gestational age (SGA), congenital malformation, admission to a neonatal intensive care unit, birth weight, Apgar scores, neonatal 25-hydroxyvitamin D (25[OH]D) and calcium concentrations, gestational age, preterm birth, infant anthropometry, and respiratory morbidity during childhood.

**Results:**

Twenty-four clinical trials involving 5405 participants met inclusion criteria. Vitamin D supplementation during pregnancy was associated with a lower risk of SGA (RR, 0.72; 95% CI, 0.52 to 0.99; RD, −5.60%; 95% CI, −0.86% to −10.34%) without risk of fetal or neonatal mortality (RR, 0.72; 95% CI, 0.47 to 1.11) or congenital abnormality (RR, 0.94; 95% CI, 0.61 to 1.43). Neonates with prenatal vitamin D supplementation had higher 25(OH)D levels (MD, 13.50 ng/mL; 95% CI, 10.12 to 16.87 ng/mL), calcium levels (MD, 0.19 mg/dL; 95% CI, 0.003 to 0.38 mg/dL), and weight at birth (MD, 75.38 g; 95% CI, 22.88 to 127.88 g), 3 months (MD, 0.21 kg; 95% CI, 0.13 to 0.28 kg), 6 months (MD, 0.46 kg; 95% CI, 0.33 to 0.58 kg), 9 months (MD, 0.50 kg; 95% CI, 0.01 to 0.99 kg), and 12 months (MD, 0.32 kg; 95% CI, 0.12 to 0.52 kg). Subgroup analysis by doses showed that low-dose vitamin D supplementation (≤2000 IU/d) was associated with a reduced risk of fetal or neonatal mortality (RR, 0.35; 95% CI, 0.15 to 0.80), but higher doses (>2000 IU/d) did not reduce this risk (RR, 0.95; 95% CI, 0.59 to 1.54).

**Conclusions and Relevance:**

Vitamin D supplementation during pregnancy is associated with a reduced risk of SGA and improved infant growth without risk of fetal or neonatal mortality or congenital abnormality. Vitamin D supplementation with doses of 2000 IU/d or lower during pregnancy may reduce the risk of fetal or neonatal mortality.

## Key Points

**Question:**

Is vitamin D supplementation during pregnancy beneficial and safe for offspring?

**Findings:**

In this systematic review and meta-analysis of 24 randomized clinical trials including 5405 individuals, vitamin D supplementation during pregnancy was associated with a lower risk of infants being small for gestational age and improved growth during infancy without an increased risk of fetal or neonatal mortality or congenital abnormality.

**Meaning:**

Vitamin D supplementation during pregnancy may reduce the risk of infants being small for gestational age and improve growth during infancy without an increased risk of fetal or neonatal mortality or congenital abnormality.

## Teaser

This systematic review and meta-analysis assesses findings from clinical trials that evaluated the association of vitamin D supplementation during pregnancy with outcomes in the fetus and neonate.

## Introduction

Low maternal vitamin D level status is common during pregnancy and is a public health issue worldwide.^1,2,3^ Vitamin D, a fat-soluble nutrient and prohormone,^2^ has classic functions of calcium absorption, metabolism, and bone health and nonclassic actions that may affect various other aspects of health.^4^ Low vitamin D level status during pregnancy may expose the offspring to a suboptimal nutritional environment during critical phases of fetal development and may have long-term effects on offspring health outcomes.^3,5,6^ Sufficient vitamin D concentrations are needed during pregnancy to address the increased demand of fetal growth and development because the mother provides all of the vitamin D for the fetus.^7^

During the past few decades, emerging randomized clinical trials (RCTs) have assessed the effect of vitamin D supplementation during pregnancy on maternal, neonatal, infant, or child outcomes. However, the results of the RCTs are inconsistent.^2^ There is a lack of evidence from systematic reviews and meta-analyses to evaluate the association between vitamin D supplementation during pregnancy and offspring growth, morbidity, and mortality.^4^ Given the high prevalence of low vitamin D level status during pregnancy and the public health importance of clarifying the role of vitamin D during pregnancy in offspring health, we conducted a systematic review and meta-analysis of RCTs with aims to evaluate the effectiveness and safety of vitamin D supplementation during pregnancy on offspring outcomes.

## Methods

### Data Sources and Searches

This systematic review is presented according to Preferred Reporting Items for Systematic Reviews and Meta-analyses (PRISMA) guidelines.^8^ Medline, Embase, and the Cochrane Database of Systematic Reviews were searched up to October 31, 2017. The key words used were *vitamin D*, *pregnancy*, *randomized controlled trials*, and *offspring outcomes*. References cited in these articles were manually searched to identify additional RCTs.

### Study Selection

Two investigators (W.G.B. and S.Q.W.) independently scrutinized the electronic searches and obtained full articles of all citations that were potentially eligible studies for inclusion. Full-length articles of studies evaluating maternal vitamin D supplementation in pregnancy and offspring outcomes were examined and subsequently selected if they fulfilled the following inclusion criteria: (1) the design was an RCT; (2) population was healthy, pregnant women without prior vitamin D supplementation of more than 400 IU/d; (3) vitamin D protocol was specified in the treatment group; (4) outcomes were offspring growth, morbidity, and mortality; (5) the study contained relevant data to calculate the effect size; and (6) the study met the methodologic quality assessment criteria for RCTs.^9^ Articles were excluded if (1) they were reviews, observational studies, case reports, letters, or comments; (2) there was no appropriate control group; (3) vitamin D dose in the intervention group was 400 IU/d or less; or (4) data were incomplete or conflicting.

Primary outcomes were (1) small for gestational age (SGA), indicated by birth weight less than the 10th percentile for gestational age, and (2) fetal or neonatal mortality. Secondary outcomes were (1) neonatal 25-hydroxyvitamin D (25[OH]D) levels, (2) congenital malformation, (3) admission to a neonatal intensive care unit (NICU), (4) Apgar scores, (5) neonatal calcium levels, (6) birth weight, (7) low birth weight, (8) gestational age, (9) preterm birth, (10) infant growth, (11) asthma, (12) respiratory infection, (13) eczema, and (14) allergy.

### Quality Assessment

We evaluated the methodologic quality of each eligible RCT using the Cochrane Risk Assessment Tool (eTable in the Supplement).^9^ The following items were evaluated: random sequence generation (selection bias), allocation concealment (selection bias), blinding of participants and personnel (performance bias), blinding of outcome assessment (detection bias), incomplete outcome data (attrition bias), selective reporting (reporting bias), and other biases. For all RCTs, each item was described as having a low risk of bias, a high risk of bias, or an unclear risk of bias.^9^

### Data Extraction and Synthesis

The following information was extracted from the study reports: the first author’s last name, year of publication, country of origin, study design, total sample size, characteristics of participants, timing of supplementation, interventions, and outcomes. When the study had 2 or more intervention groups with different doses of vitamin D supplementation, we combined them into 1 intervention group. Two of us (W.G.B. and S.Q.W.) extracted the data independently and in duplicate. Discrepancies were resolved through discussion to achieve a consensus.

Subgroup analyses were performed according to timing (initiation at <20 or ≥20 weeks’ gestation), dose (>2000 or ≤2000 IU/d), and method (regular or bolus doses) of vitamin D supplementation for the outcomes of SGA, fetal or neonatal mortality, neonatal blood 25(OH)D concentration, and birth weight.

### Statistical Analysis

Data on dichotomous outcomes were combined using the Mantel-Haenszel method, and measures of effect are presented as risk ratios (RRs) or risk differences with 95% CIs. For continuous data, we calculated the sample size–weighted mean difference (MD) when outcomes were measured in the same way between studies. We used forest plots to show the point estimate (95% CIs) for each study. The *I*^2^ statistic (percentage of variability in the results that is due to heterogeneity) was used to quantify the degree of heterogeneity across studies.^10^ If the *I*^2^ value was 50% or greater, the heterogeneity was considered significant and we pooled results using a random-effects model. Otherwise, a fixed-effect model was applied. Funnel plots were applied to evaluate publication bias. The data were extracted and statistical analysis was carried out using Review Manager, version 5.3 (RevMan).^11^ Two-tailed *P* < .05 values were considered statistically significant.

## Results

### Study Selection

**Figure 1.**
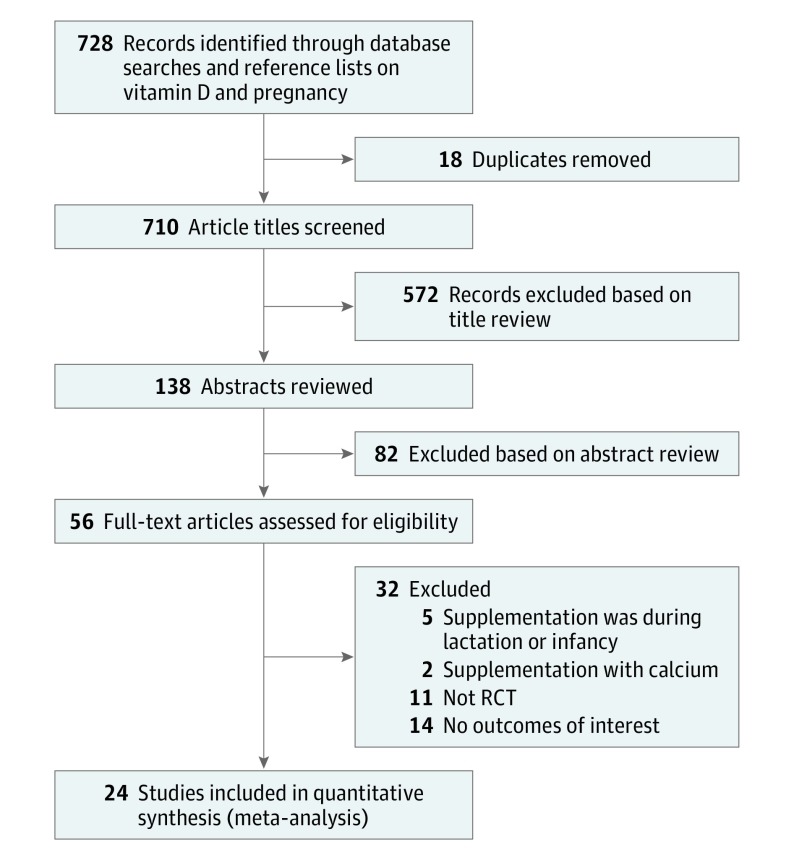
Flowchart of Study Selection Process RCT indicates randomized clinical trial.

The search strategy resulted in 728 potentially relevant citations. The PRISMA flow diagram (Figure 1) summarizes the process of the literature search and selection of studies. After screening the titles and abstracts, we read 56 articles. Twenty-four RCTs^12,13,14,15,16,17,18,19,20,21,22,23,24,25,26,27,28,29,30,31,32,33,34,35,36^ comprising 5405 participants met the inclusion criteria. Two of these trials are from the same RCT with different outcomes.^17,36^ The assessment of methodologic quality of each eligible RCT by the Cochrane Risk Assessment Tool is summarized in the eTable in the Supplement.

### Study Characteristics

The characteristics of the included RCTs are summarized in the Table. Vitamin D supplementation was in the form of cholecalciferol in 22 RCTs^13,14,15,16,17,18,19,20,21,22,23,24,26,27,28,29,30,31,32,33,34,35^ and in the form of ergocalciferol in 3 RCTs.^12,17,25^ For the intervention group, the daily doses were 800 IU in 1 RCT,^17^ 1000 IU in 6 RCTs,^12,14,16,18,25,26^ 1200 IU in 1 RCT,^34^ 2000 IU in 7 RCTs,^15,18,20,26,29,34,35^ 2800 IU in 1 RCT,^13^ 4000 IU in 4 RCTs,^15,20,21,29^ 4400 in 1 RCT,^24^ or 5000 IU in 1 RCT^33^; the weekly doses were 35 000 IU^30^ or 50 000 IU^19^; the fortnightly dose was 50 000 IU in 2 RCTs^23,28^; the monthly dose was 60 000 IU^32^; the bimonthly dose was 60 000 IU^32^; and the bolus doses were 60 000 IU in 3 RCTs,^22,27,31^ 120 000 IU in 2 RCTs,^22,31^ or 200 000 IU in 2 RCTs.^17,25^ In studies comprising 3 or more groups, as was the case in 9 RCTs,^15,17,18,20,22,25,26,32,34^ the higher-dose groups were combined into 1 cohort as the intervention group and the lowest dose groups as the control cohort. There were no toxic effects on offspring in the included RCTs and no evidence of publication bias.

**Table.** Characteristics of Included Randomized Clinical Trials

Source	Country	Total Sample Size	Participants	Initiation and Timing of Supplementation	Interventions	Outcomes
Brooke et al,^12^ 1980	United Kingdom	126	Pregnant Asian women	Third trimester	Ergocalciferol, 1000 IU/d, vs placebo	Cord blood 25(OH)D concentration, neonatal anthropometry, SGA, LBW, gestational age at birth, anthropometry at birth, 3, 6, 9, and 12 mo
Chawes et al,^13^ 2016	Denmark	623	Pregnant women not after wk 26; without endocrine, cardiovascular, or nephrologic disorders; vitamin D_3_ intake no more than 600 IU/d	24 wk of gestation to 1 wk postpartum	Cholecalciferol, 2800 IU/d, vs 400 IU/d	Fetal or neonatal death, congenital malformation, admission to a NICU, preterm birth, wheeze, asthma, upper and lower respiratory tract infections, eczema, allergy skin prick test, allergy-specific IgE at age 3 y
Cooper et al,^14^ 2016	United Kingdom	965	Pregnant women >18 y, singleton pregnancy, gestation <17 weeks, serum 25(OHD) level 10-40 ng/mL at 10-17 wk of gestation	14 wk of gestation or as soon as possible before 17 wk of gestation if recruited later until delivery	Cholecalciferol, 1000 IU/d, vs placebo	Fetal or neonatal death, congenital malformation, neonatal anthropometry, preterm birth
Dawodu et al,^15^ 2013	United States	126	Arab expectant mothers, 12-16 wk of gestation, singleton pregnancy	12-16 wk of gestation until delivery	Cholecalciferol, 2000 or 4000 IU/d, vs 400 IU/d	Cord blood 25(OH)D concentration, SGA
Delvin et al,^16^ 1986	France	30	Pregnant women	Third trimester	Cholecalciferol, 1000 IU/d, vs no treatment	Cord blood 25(OH)D concentration
Goldring et al,^17^ 2013; Yu et al,^36^ 2009	United Kingdom	179	Pregnant women	27 wk of gestation until delivery	Ergocalciferol, 800 IU/d, or cholecalciferol, 200 000 IU (1 dose), vs no treatment (control)	Fetal or neonatal death, cord blood 25(OH)D concentration, neonatal anthropometry, SGA, gestational age at birth, wheeze, eczema, upper and lower respiratory tract infections at age 3 y
Grant et al,^18^ 2014	New Zealand	258	Pregnant women, 26-30 wk of gestation, singleton pregnancy, no vitamin D supplementation >200 IU/d, history of renal stones, hypercalcemia, or any serious pregnancy complication at enrollment	27 wk of gestation until delivery	Cholecalciferol, 1000 or 2000 IU/d, vs placebo	Fetal or neonatal death, cord blood 25(OH)D concentration, preterm birth, asthma, upper and lower respiratory tract infections, allergy skin prick test, allergy-specific IgE at age 3 y
Hashemipour et al,^19^ 2014	Iran	110	Iranian pregnant women with vitamin D deficiency	Start at 26-28 wk of gestation; duration, 8 wk	Cholecalciferol, 50 000 IU/wk, vs 400 IU/d	Fetal or neonatal death, cord blood 25(OH)D concentration, neonatal anthropometry, SGA, preterm birth
Hollis et al,^20^ 2011	United States	350	Women with a singleton pregnancy	12-16 wk of gestation until delivery	Cholecalciferol, 2000 or 4000 IU/d, vs 400 IU/d	Fetal or neonatal death, admission to a NICU, cord blood 25(OH)D concentration, neonatal anthropometry, gestational age at birth
Hossain et al,^21^ 2014	Pakistan	175	Women with singleton pregnancy	20 wk of gestation until delivery	Cholecalciferol, 4000 IU/d, vs routine care	Fetal or neonatal death, Apgar score, cord blood 25(OH)D concentration, neonatal anthropometry, SGA, gestational age at birth, preterm birth
Kalra et al,^22^ 2012	Zimbabwe	109	Pregnant women	12-24 wk of gestation until delivery	Cholecalciferol, 120 000 IU (1 dose) or 60 000 IU (2 doses), vs standard care	Neonatal anthropometry, infant anthropometry at 3, 6, and 9 mo
Karamali et al,^23^ 2015	Iran	60	Pregnant women prima gravida, aged 18-40 y, at risk for preeclampsia, without abnormal fetal anomaly scan	20-30 wk of gestation	Cholecalciferol, 50 000 IU/fortnight, vs placebo	Apgar score, neonatal anthropometry, LBW, gestational age at birth, preterm birth
Litonjua et al,^24^ 2016	United States	835	Pregnant women aged 18-39 y; gestational age 10-18 wk; history of asthma, eczema, or allergic rhinitis; nonsmoker; English or Spanish speaking	10-18 wk of gestation until delivery	Cholecalciferol, 4400 vs 400 IU/d	Fetal or neonatal death, congenital malformation, admission to a NICU, cord blood 25(OH)D concentration, neonatal anthropometry, preterm birth, asthma, lower respiratory tract infections, eczema, allergy skin prick test, allergy-specific IgE at age 3 y
Mallet et al,^25^ 1986	France	77	White pregnant women	7 mo of gestation	Ergocalciferol, 1000 IU/d or 200 000 IU (1 dose), vs control	Cord blood 25(OH)D concentration
March et al,^26^ 2015	Canada	105	Pregnant women aged 18-45 y, healthy, 13-24 wk of gestation, exclusion of women receiving supplements >400 IU/d	13-24 wk of gestation until delivery	Cholecalciferol, 1000 or 2000 IU/d, vs 400 IU/d	Cord blood 25(OH)D concentration
Marya et al,^27^ 1988	India	200	Pregnant women aged 22-35 y	7 mo of gestation	Cholecalciferol, 600 000 IU (2 doses), vs no supplementation	Neonatal anthropometry, LBW, gestational age at birth
Mojibian et al,^28^ 2015	Iran	389	Pregnant women, 12-16 wk of gestation, serum 25(OH)D <30 ng/mL	12 wk of gestation until delivery	Cholecalciferol, 50 000 IU/fortnight, vs 400 IU/d	Apgar score, cord blood 25(OH)D concentration, neonatal anthropometry, LBW, preterm birth
Rodda et al,^29^ 2015	Australia	45	Pregnant women, singleton pregnancy, serum 25(OH)D <30 ng/mL	12-16 wk of gestation until delivery	Cholecalciferol, 2000 IU/d (adjusted to 4000 IU/d if serum vitamin D level remains <75 nmol/L), vs standard care	Cord blood 25(OH)D concentration
Roth et al,^30^ 2013	United States	147	Pregnant women	Third trimester	Cholecalciferol, 35 000 IU/wk, vs placebo	Fetal or neonatal death, 25(OH)D concentration, neonatal anthropometry, gestational age at birth, preterm birth, infant anthropometry at 12 mo, weight, length, and head circumference *z* scores in infants at age 1 y
Sablok et al,^31^ 2015	India	165	Prima gravida with singleton pregnancy at 14-20 wk, without preexisting osteomalacia, known hyperparathyroidism, renal or liver dysfunction, tuberculosis, or sarcoidosis	20 wk of gestation until delivery	Cholecalciferol, 60 000 IU (1 dose), 120 000 IU (2 doses), or 120 000 IU (4 doses), vs no supplementation	Cord blood 25(OH)D concentration, neonatal anthropometry, SGA, preterm birth
Sahoo et al,^32^ 2017	India	52	Pregnant women aged >18 y, singleton pregnancy, <20 wk of gestation, no known bone diseases or complicated pregnancy, no vitamin D supplementation within previous 3 mo	14-20 wk of gestation until delivery	Cholecalciferol, 60 000 IU/4 wk or 60 000 IU/8 wk, vs 400 IU/d	Cord blood 25(OH)D concentration, neonatal anthropometry, weight, length, and head circumference *z* scores in infants at age 1 y
Yap et al,^33^ 2014	Australia	179	Women with singleton pregnancies, aged ≥18 y and gestational age <20 wk, no history of diabetes, calcium or vitamin D metabolism disorders, hypercalcemia, or significant renal impairment, no vitamin D supplements ≥1000 IU/d	20 wk of gestation until delivery	Cholecalciferol, 5000 IU/d, vs 400 IU/d	Fetal or neonatal death, cord blood 25(OH)D concentration, neonatal anthropometry, gestational age at birth, preterm birth
Yesiltepe Mutlu et al,^34^ 2014	Turkey	51	Pregnant women aged >16 y, singleton pregnancy, no previously known calcium metabolism or untreated thyroid disorders	13-32 wk of gestation until delivery	Cholecalciferol, 1200 or 2000 IU/d, vs 600 IU/d	Neonatal anthropometry, neonatal 25(OH)D concentration
Zerofsky et al,^35^ 2016	United States	49	Participants aged >18 y with a singleton pregnancy <20 wk	No later than 20 wk of gestation until delivery	Cholecalciferol, 2000 IU/d, vs 400 IU/d	Apgar score, neonatal anthropometry, gestational age at birth

Abbreviations: IgE, immunoglobulin E; LBW, low birth weight; NICU, neonatal intensive care unit; RCT, randomized clinical trial; SGA, small for gestational age; 25(OH)D, 25-hydroxyvitamin D.

SI conversion factor: To convert 25(OH)D to nanomoles per liter, multiply by 2.496.

**Figure 2.**
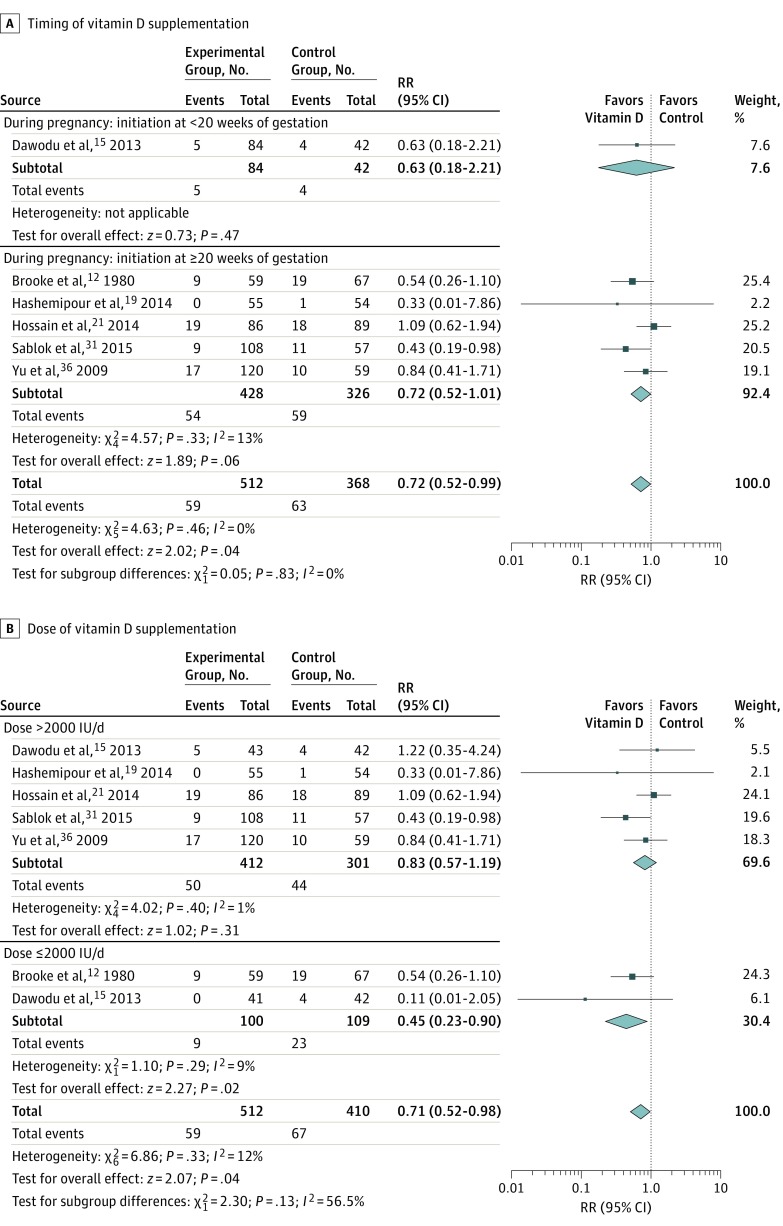
Summary Risk Ratio (RR) of the Association Between Vitamin D Supplementation and Small for Gestational Age (SGA) Subgroup analyses by timing (initiation at <20 or ≥20 weeks of gestation) (A) and dose (>2000 or ≤2000 IU/d) (B). Diamond at the bottom represents the pooled point estimate (95% CIs) for each outcome of interest.

The vitamin D supplementation group had a significantly lower risk of SGA (RR, 0.72; 95% CI, 0.52 to 0.99; *I*^2^ = 0%) in 6 RCTs^12,15,19,21,31,36^ with 898 participants (Figure 2). Risk difference was −5.60%; 95% CI, −0.86% to −10.34%. Subgroup analysis by doses showed that vitamin D supplementation at 2000 IU/d or lower was associated with a reduced risk of SGA (RR, 0.45; 95% CI, 0.23 to 0.90), while vitamin D supplementation at doses larger than 2000 IU/d was not associated with a reduced risk of SGA (RR, 0.83; 95% CI, 0.57 to 1.19). Testing of subgroups showed no significant differences, but significant heterogeneity was present (*P* = .13; *I*^2^ = 56.5%) (Figure 2B). Timing (early or late) (Figure 2A) and method (regular or bolus doses) (eFigure 1 in the Supplement) of vitamin D supplementation had no association with the risk of SGA.

**Figure 3.**
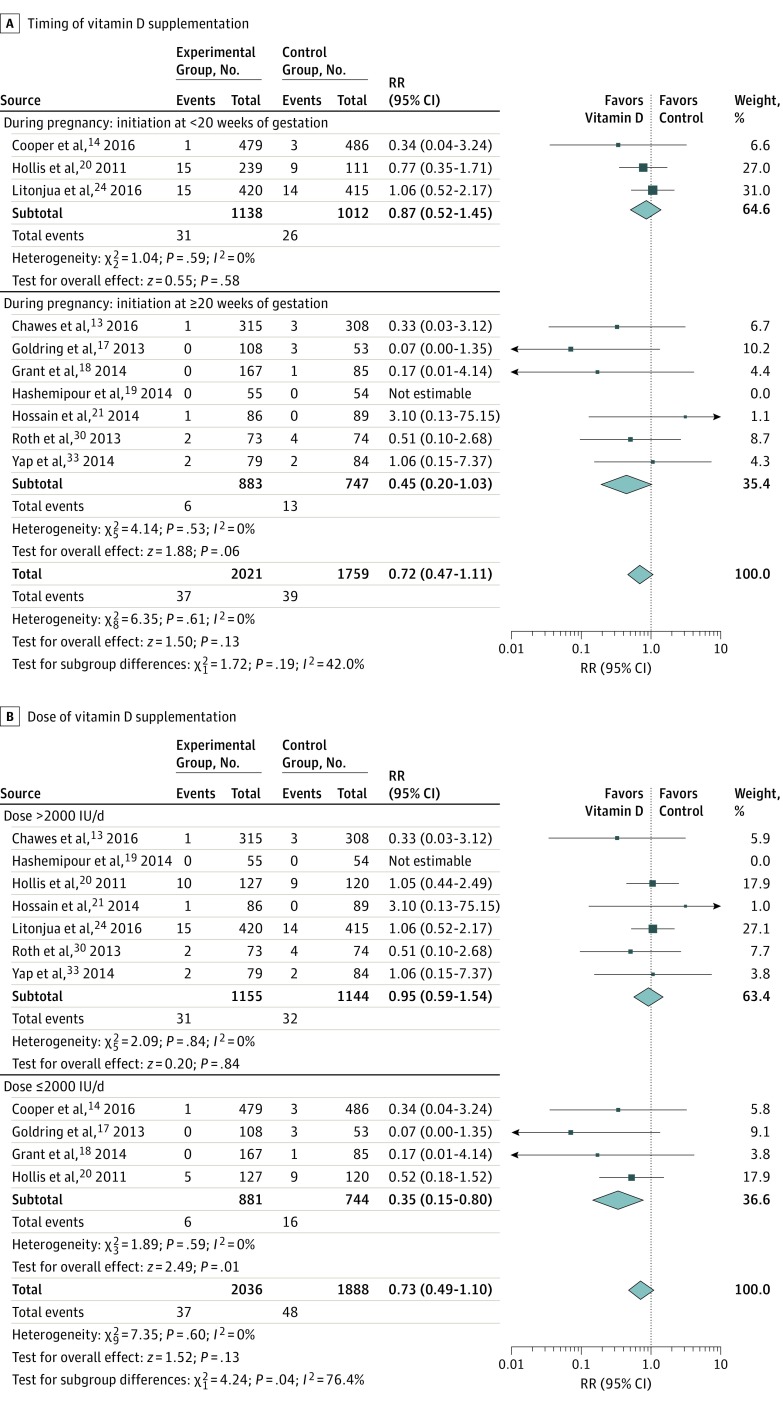
Summary Risk Ratio (RR) of the Association Between Vitamin D Supplementation and Fetal or Neonatal Mortality Subgroup analyses by timing (initiation at <20 or ≥20 weeks of gestation) (A) and dose (>2000 or ≤2000 IU/d) (B). Diamond at the bottom represents the pooled point estimate (95% CIs) for each outcome of interest.

Vitamin D supplementation during pregnancy was not associated with a risk of fetal or neonatal mortality (RR, 0.72; 95% CI, 0.47-1.11; *I*^2^ = 0%) in 10 RCTs^13,14,17,18,19,20,21,24,30,33^ with 3780 participants (Figure 3). Subgroup analysis by doses showed that vitamin D supplementation at 2000 IU/d or less was associated with reduced risk of fetal or neonatal mortality (RR, 0.35; 95% CI, 0.15-0.80), while vitamin D supplementation at doses larger than 2000 IU/d did not reduce the risk of fetal or neonatal mortality (RR, 0.95; 95% CI, 0.59-1.54). Testing for subgroup difference was statistically significant (RR, 0.73; 95% CI, 0.49-1.10; *P* = .04) (Figure 3B). Timing (early or late) (Figure 3A) and method (regular or bolus doses) (eFigure 2 in the Supplement) of vitamin D supplementation had no association with the risk of fetal or neonatal mortality.

There was no significant difference between neonates who received prenatal vitamin D supplementation and those who had not in the outcomes of congenital malformation (RR, 0.94; 95% CI, 0.61-1.43; *I*^2^ = 0%) in 3 RCTs^13,14,24^ with 2355 participants and admission to a NICU (RR, 1.11; 95% CI, 0.82-1.51; *I*^2^ = 0%) in 3 RCTs^13,20,24^ with 1740 participants; however, the supplementation group had significantly higher Apgar scores at 1 minute (MD, 0.09; 95% CI, 0.01-0.17; *I*^2^ = 40%) in 4 RCTs^21,23,28,35^ with 670 participants and at 5 minutes (MD, 0.08; 95% CI, 0.02-0.14; *I*^2^ = 13%) in 4 RCTs^21,23,28,35^ with 668 participants.

Results show that, compared with the control group, the vitamin D supplementation group had higher 25(OH)D concentrations (MD, 13.50 ng/mL; 95% CI, 10.12-16.87 ng/mL; *I*^2^ = 97%) in 14 RCTs^12,16,17,19,20,24,25,26,28,29,30,32,33,34^ with 2361 participants and had more neonates achieving levels of 20 ng/mL (RR, 2.81; 95% CI, 1.92-4.12; *I*^2^ = 68%) in 7 RCTs^15,17,18,20,30,31,34^ with 1107 participants or 30 ng/mL (RR, 5.20; 95% CI, 3.34-8.10; *I*^2^ = 47%) in 3 RCTs^18,19,21^ with 485 participants. Subgroup analysis for neonatal 25(OH)D concentrations by supplementation timing, dose, or method showed that vitamin D supplementation increased neonatal blood 25(OH)D levels whether the supplementation was initiated early (<20 weeks’ gestation) or late (≥20 weeks’ gestation), at higher doses (>2000 IU/d) or lower doses (≤2000 IU/d), or as bolus or regular doses (eFigure 3 in the Supplement).

Neonates who received prenatal vitamin D supplementation had higher calcium concentrations (to convert to millimoles per liter, multiply by 0.25) than those who received no intervention or placebo (MD, 0.19 mg/dL; 95% CI, 0.003-0.38 mg/dL; *I*^2^ = 74%) in 9 RCTs^12,16,18,22,25,27,32,33,34^ with 1007 participants) (eFigure 4 in the Supplement).

Neonates who received prenatal vitamin D supplementation had significantly greater birth weight (MD, 75.38 g; 95% CI, 22.88 to 127.88 g; *I*^2^ = 44%) in 17 RCTs^12,14,17,19,20,21,22,23,24,27,28,30,31,32,33,34,35^ with 4087 participants, greater neonatal femur length (MD, 0.12 cm; 95% CI, 0.01 to 0.23 cm; *I*^2^ = 0%) in 2 RCTs^30,33^ with 316 participants, and greater skinfold thickness (MD, 0.34 mm; 95% CI, 0.17 to 0.51 mm; *I*^2^ = 34%) in 2 RCTs^12,27^ with 326 participants, but no significant difference was observed for crown heel length (MD, 0.33 cm; 95% CI, −0.05 to 0.70 cm; *I*^2^ = 74%) in 12 RCTs^12,14,19,21,22,23,24,27,28,30,32,33^ with 3301 participants or head circumference (MD, 0.20 cm; 95% CI, −0.04 to 0.43 cm; *I*^2^ = 78%) in 11 RCTs^12,14,19,21,22,23,24,27,28,30,33^ with 3240 participants. Subgroup analysis by supplementation timing showed that vitamin D supplementation increased birth weight only in the group with therapy initiated late (≥20 weeks’ gestation) (MD 97.74 g; 95% CI, 29.40 to 166.08 g). Test for subgroup differences between early and late supplementation showed significant differences (χ^2^ = 5.91_1_; *P* = .02; *I*^2^ = 83.1%) (eFigure 5A in the Supplement). Test for subgroup differences by dose showed no significant difference between higher dose and lower dose (χ^2^ = 0.13_1_; *P* = .72; *I*^2^ = 0%) (eFigure 5B in the Supplement). Test for subgroup differences by supplementation method showed no significant difference in effect between regular and bolus dose (χ^2^ = 0.07_1_; *P* = .79; *I*^2^ = 0%) (eFigure 5C in the Supplement).

There was no significant difference between neonates who received prenatal vitamin D supplementation and those who had not in the outcomes of low birth weight (RR, 0.52; 95% CI, 0.20 to 1.37; *I*^2^ = 65%) in 4 RCTs^12,23,27,28^ with 775 participants, gestational age (MD, −0.08 weeks; 95% CI, −0.68 to 0.53 weeks; *I*^2^ = 81%) in 9 RCTs^12,17,20,21,23,27,30,33,35^ with 1441 participants, or preterm birth (RR, 0.98; 95% CI, 0.77 to 1.26; *I*^2^ = 33%) in 11 RCTs^13,14,18,19,21,23,24,28,30,31,33^ with 3822 participants).

On infant anthropometry, 2 RCTs^12,22^ reported on outcomes at 3 months (216 participants), 6 months (199 participants), and 9 months (179 participants), and 2 RCTs^12,30^ reported at 12 months. Results showed that infants who received prenatal vitamin D supplementation had significantly greater weight at 3 months (MD, 0.21 kg; 95% CI, 0.13 to 0.28 kg; *I*^2^ = 0%) (eFigure 6A in the Supplement), 6 months (MD, 0.46 kg; 95% CI, 0.33 to 0.58 kg; *I*^2^ = 0%) (eFigure 6B in the Supplement), 9 months (MD, 0.50 kg; 95% CI, 0.01 to 0.99 kg; *I*^2^ = 89%) (eFigure 6C in the Supplement), and 12 months (MD, 0.32 kg; 95% CI, 0.12 to 0.52 kg; *I*^2^ = 47%; 252 participants) (eFigure 6D in the Supplement); significantly greater height at 3 months (MD, 1.09 cm; 95% CI, 0.64 to 1.54; cm; *I*^2^ = 16%), 9 months (MD, 1.47 cm; 95% CI, 0.13 to 2.82 cm; *I*^2^ = 80%), and 12 months (MD, 1.36 cm; 95% CI, 0.81 to 1.92 cm; *I*^2^ = 40%; 251 participants) but not at 6 months (MD, 1.35 cm; 95% CI, −0.30 to 3.00 cm; *I*^2^ = 87%); and significantly greater head circumference at 3 months (MD, 0.71 cm; 95% CI, 0.23 to 1.18 cm; *I^2^* = 64%) but not at 6 months (MD, 0.54 cm; 95% CI, −0.04 to 1.13 cm; *I*^2^ = 72%), 9 months (MD, 0.36 cm; 95% CI, −0.16 to 0.88 cm; *I*^2^ = 15%), or 12 months (MD, 0.09 cm; 95% CI, −0.28 to 0.45 cm; *I*^2^ = 0%; 248 participants).

Vitamin D supplementation showed no association with the infants’ outcomes of asthma (RR, 0.63; 95% CI, 0.36-1.11; *I*^2^ = 71%) in 3 RCTs^13,18,24^ with 1591 participants, eczema (RR, 0.92; 95% CI, 0.77-1.11; *I*^2^ = 0%) in 3 RCTs^13,17,24^ with 1538 participants, upper respiratory tract infection (RR, 0.94; 95% CI, 0.79-1.12; *I*^2^ = 27%) in 2 RCTs^17,18^ with 389 participants, lower respiratory tract infection (RR, 0.97; 95% CI, 0.85-1.12; *I*^2^ = 0%) in 4 RCTs^13,17,18,24^ with 1769 participants, allergy skin prick test (RR, 0.88; 95% CI, 0.52-1.49; *I*^2^ = 60%) in 3 RCTs^13,18,24^ with 1304 participants, or presence of allergy-specific immunoglobulin E (RR, 0.80; 95% CI, 0.39-1.67; *I*^2^ = 78%) in 3 RCTs^13,18,24^ with 1298 participants. The funnel plots for the primary outcomes showed no publication bias in SGA and fetal or neonatal mortality (eFigure 7 in the Supplement).

## Discussion

The main finding of this systematic review and meta-analysis of RCTs was that vitamin D supplementation during pregnancy was associated with a reduced risk of SGA (RR, 0.72) without an increased risk of fetal or neonatal mortality and congenital malformation. Vitamin D supplementation during pregnancy with lower doses (≤2000 IU/d) was associated with a reduced risk of fetal and neonatal mortality. Vitamin D supplementation was associated with higher neonatal vitamin D status (bolus- or regular-dose supplement and early or late timing were equally effective in attaining improvement in vitamin D levels), higher calcium levels, higher Apgar scores, greater neonatal skinfold thickness, greater weight (at birth, 3 months, 6 months, 9 months, and 12 months), and greater height (at 3 months, 9 months, and 12 months) in the offspring. Timing of vitamin D supplementation affected birth weight. There was no significant difference in the offspring outcomes of gestational age, preterm birth, asthma, eczema, respiratory tract infection, or allergy. Based on the results from this meta-analysis, the number needed to treat for SGA was 18: 1 offspring SGA case could be avoided for every 18 pregnant women receiving vitamin D supplementation during pregnancy.

The quality of systematic reviews depends on the quality of the studies included. We evaluated the risk of bias in the RCTs analyzed. Methodologic issues may affect the study quality. We scrutinized the selected studies of good methodologic quality using strict quality assessment criteria.^9^ Our systematic review is a comprehensive quantitative review of 24 RCTs that reported the effects of maternal vitamin D supplementation in offspring health outcomes, including SGA, fetal or neonatal mortality, congenital malformation, admission to a NICU, Apgar scores, neonatal 25(OH)D and calcium concentrations, preterm birth, anthropometric indicators (weight, height, head circumference, or skinfold thickness) during infancy (at birth and ages 3, 6, 9, and 12 months), asthma, eczema, respiratory tract infection, and allergy in the first 3 years of life.

Previous systematic reviews^3,37,38^ reported that vitamin D supplementation during pregnancy increased maternal 25(OH)D levels^3,37^ or neonatal 25(OH)D concentrations.^38^ One systematic review^3^ evaluated the outcome of vitamin D supplementation during pregnancy for maternal 25(OH)D levels, risk of preeclampsia, gestational diabetes, and other maternal complications but lacked review on offspring outcomes. A Cochrane review^39^ studied the association between supplementing vitamin D in pregnant women alone or in combination with calcium along with maternal complications and neonatal outcomes and showed no association between vitamin D supplementation and birth weight in 5 RCTs. Another systematic review^40^ assessed maternal and neonatal outcomes and showed that birth weight in 8 RCTs and length in 6 RCTs were greater in the vitamin D supplementation group; however, this review had no information on infant follow-up.

The present review adds to the existing literature by including a greater number of recent RCTs and, to our knowledge, is the first meta-analysis of RCTs reporting that vitamin D supplementation during pregnancy was safe (without increased risk of fetal or neonatal mortality, congenital abnormality, or admission to a NICU) and effective in reducing the risk of SGA and improving neonatal calcium levels, skinfold thickness, and postnatal growth (greater weight and height at ages 3, 6, 9, or 12 months). We found that maternal vitamin D supplementation timing, dose, and administration method did not affect cord blood vitamin D concentration. Late vitamin D supplementation (initiation at ≥20 weeks’ gestation) improved birth weight, but early supplementation (initiation at <20 weeks’ gestation) did not. Most importantly, we found that the lower dose of vitamin D supplementation (≤2000 IU/d) reduced the risk of fetal or neonatal mortality and SGA, but the higher dose (>2000 IU/d) did not.

Our findings that maternal vitamin D supplementation during pregnancy reduced the risk of SGA and improved infant growth are biologically plausible. Maternal vitamin D levels during pregnancy positively affect infant bone formation^41^ as well as skeletal muscle^42^ and adiposity development,^43^ which are important for infant growth and development. Vitamin D is needed in maintaining normal levels of calcium and phosphate in blood, which in turn facilitate the process of mineral ion homeostasis and bone formation during early life.^44^ Increased maternal vitamin D status improved fetal skeletal muscle development and myoblast activity.^42^ Members of the Southampton Developmental Origins of Health and Disease research group reported that low maternal vitamin D status at 34 weeks’ gestation was associated with lower fat mass at birth.^43^ Vitamin D also plays an important role in the modulation of the immune function^45^ and oxidative stress^46^ that may link to fetal growth. In addition, vitamin D regulates genes responsible for trophoblast invasion and angiogenesis critical for placental implantation and function,^47,48,49^ which is important for fetal growth.

Vitamin D during pregnancy has been linked to fetal lung maturation in animal models.^50,51^ Maternal vitamin D may exert its influence during pregnancy on the respiratory and immune systems during lung development in early childhood.^52^ However, the results of this meta-analysis show that vitamin D supplementation during pregnancy was not associated with childhood respiratory or immune outcomes, including upper or lower respiratory tract infections, asthma, eczema, or allergy, in children at age 3 years. Christensen et al^53^ conducted a meta-analysis of maternal vitamin D supplementation during pregnancy and infant respiratory tract infections; their results were in line with ours with respect to respiratory tract infections, but they did not have results on asthma. Long-term follow-up of children is needed to determine the effect of vitamin D supplementation during pregnancy on other health outcomes.

### Limitations

This study has limitations. First, there were limited data on maternal vitamin D supplementation during pregnancy regarding long-term offspring outcomes, and the longest follow-up in the included RCTs was 3 years. Second, there were only 2 studies on the outcomes of infant growth at age 3 months,^12,22^ 6 months,^12,22^ 9 months,^12,22^ and 12 months^12,30^; this result has to be interpreted cautiously. In addition, there was heterogeneity in the result of weight in infants at age 9 months; it is not clear why these 2 studies show different patterns in infants at this age. From a developmental perspective, at 9 months, infants’ weight may differ because of transition to solid food and the total intake, and some infants will begin to walk. Third, the included RCTs differed in several aspects, such as the population studied, ethnicity, altitude, latitude, the outcomes chosen, the clinical setting, the timing of the intervention, and the dose of vitamin D administered during pregnancy. Fourth, the variability in the assay methods for 25(OH)D measurement in each study may contribute to the heterogeneity of the neonatal vitamin D levels. Finally, there were limited data on adherence to the respective protocols.

## Conclusions

Vitamin D supplementation during pregnancy was associated with reduced risk of SGA, improved infant growth, and no risk of fetal or neonatal mortality and congenital abnormality. Vitamin D supplementation (≤2000 IU/d) during pregnancy may reduce the risk of fetal or neonatal mortality.

## Notes

Supplement. **eTable.** Assessment of Bias Risk of Randomized Clinical Trials **eFigure 1.** Summary Crude Risk Ratio (RR) of the Association Between Vitamin D Supplementation and Small for Gestational Age (SGA) Stratified by Administration Method of Intervention (Regular or Bolus Dose) **eFigure 2.** Summary Risk Ratio of the Association 1 Between Vitamin D Supplementation Group and Fetal or Neonatal Mortality Stratified by Administration Method of Intervention (Regular or Bolus Dose). **eFigure 3.** Forest Plots of Summary Mean Difference (MD) of Neonatal 25-Hydroxyvitamin D (25[OH]D) (ng/mL) Between Vitamin D Supplementation Group and Control Group **eFigure 4.** Forest Plots of Summary Mean Difference (MD) of Neonatal Calcium (mg/dL) Between Vitamin D Supplementation Group and Control Group **eFigure 5.** Forest Plots of Summary Mean Difference (MD) of Birth Weight (g) Between Vitamin D Supplementation Group and Control Group **eFigure 6.** Forest Plots of Summary Mean Difference (MD) of Postnatal Weight (kg) Between Vitamin D Supplementation Group and Control Group in Infants at Age 3 Months; 6 Months; 9 Months; and 12 Months **eFigure 7.** The Funnel Plots of the Primary Outcomes
