# Supplementary material for: Association Between Vitamin D Supplementation During Pregnancy and Offspring Growth, Morbidity, and Mortality: A Systematic Review and Meta-analysis
Source: JAMA Pediatr. 2018 May 29;172(7):635–45. doi: 10.1001/jamapediatrics.2018.0302 (PMC6137512; doi:10.1001/jamapediatrics.2018.0302)

Supplementary Online Content

Bi WG, Nuyt AM, Weiler H, Leduc L, Santamaria C, Wei SQ. Association between vitamin D supplementation during pregnancy and offspring growth, morbidity, and mortality: a systematic review and meta-analysis. *JAMA Pediatr*. Published online May 29, 2018. doi:10.1001/jamapediatrics.2018.0302

eTable. Assessment of Bias Risk of Randomized Clinical Trials

eFigure 1. Summary Risk Ratio (RR) of the Association Between Vitamin D Supplementation and Small for Gestational Age (SGA) Stratified by Administration Method of Intervention (Regular or Bolus Dose)

eFigure 2. Summary Risk Ratio of the Association Between Vitamin D Supplementation and Fetal or Neonatal Mortality Stratified by Administration Method of Intervention (Regular or Bolus Dose).

eFigure 3. Forest Plots of Summary Mean Difference (MD) of Neonatal 25-Hydroxyvitamin D (25[OH]D) (ng/mL) Between Vitamin D Supplementation Group and Control Group

eFigure 4. Forest Plots of Summary Mean Difference (MD) of Neonatal Calcium (mg/dL) Between Vitamin D Supplementation Group and Control Group

eFigure 5. Forest Plots of Summary Mean Difference (MD) of Birth Weight (g) Between Vitamin D Supplementation Group and Control Group

eFigure 6. Forest Plots of Summary Mean Difference (MD) of Postnatal Weight (kg) Between Vitamin D Supplementation Group and Control Group in Infants at Age 3 Months; 6 Months; 9 Months; and 12 Months

eFigure 7. The Funnel Plots of the Primary Outcomes

This supplementary material has been provided by the authors to give readers additional information about their work.

© 2018 Bi WG et al. *JAMA Pediatrics.*

eTable. Assessment of Bias Risk of Randomized Clinical Trials

Study	Sequence generation	Allocation concealment	Blinding of participants and personnel	Blinding of outcome assessment	Incomplete outcome data	Selective outcome data	Other sources of bias
Brooke 1980	Low	Low	Low	Low	Low	Low	Unclear
Chawes 2015	Low	Low	Low	Low	Low	Low	Unclear
Cooper 2016	Low	Low	Low	Low	Low	Low	Unclear
Dawodu 2013	Low	Low	Low	Low	Low	Low	Unclear
Delvin 1986	Unclear	Unclear	High	High	High	Low	Unclear
Goldring 2013 and Yu 2009	Low	Low	Low	Low	Low	Low	Unclear
Grant 2014	Low	Low	Low	Low	Low	Low	Unclear
Hashemipour 2014	Low	Unclear	High	Unclear	Low	Low	Unclear
Hollis 2011	Low	Unclear	Low	Low	Low	Low	Unclear
Hossain 2014	High	Unclear	High	Unclear	Low	Low	Unclear
Kalra 2012	Low	Low	Low	Low	Low	Low	Unclear
Karamal 2015	Low	Low	Low	Low	Low	Low	Unclear
Litonjua 2016	Low	Low	Low	Low	Low	Low	Unclear
Mallet 1986	Low	Low	Unclear	Unclear	Low	Low	Unclear
March 2015	Low	Low	Low	Low	High	Low	Unclear
Marya 1988	Unclear	Unclear	Unclear	Unclear	Low	Low	Unclear
Mojibian 2015	Low	Low	High	Low	Low	Low	Unclear
Rodda 2015	High	Low	High	Low	High	Low	Unclear

eTable. Assessment of Bias Risk of Randomized Clinical Trials (continued)

Roth 2013	Low	Low	Low	Low	Low	Low	Unclear
Sablok 2015	Low	High	Unclear	Unclear	Low	Low	Unclear
Sahoo 2016	Low	Low	Low	Low	High	Low	Unclear
Yap 2014	Low	Low	Low	Low	Low	Low	Unclear
Yesiltepe et Mutlu 2014	Unclear	Low	High	High	High	Low	Unclear
Zerofsky 2016	Low	Low	Low	Low	Low	Low	Unclear

eFigure 1. Summary Risk Ratio (RR) of the Association Between Vitamin D Supplementation and Small for Gestational Age (SGA) Stratified by Administration Method of Intervention (Regular or Bolus Dose).

	Experimental		Control			Risk Ratio
Study or Subgroup	Events	Total	Events	Total	Weight	M-H, Fixed, 95% CI
**1.12.1 Regular**						
Brooke 1980	9	59	19	67	23.1%	0.54 [0.26, 1.10]
Dawodu 2013	5	84	4	42	6.9%	0.63 [0.18, 2.21]
Hashemipour 2014	0	55	1	54	2.0%	0.33 [0.01, 7.86]
Hossain 2014	19	86	18	89	23.0%	1.09 [0.62, 1.94]
Yu 2009	9	60	10	59	13.1%	0.89 [0.39, 2.02]
**Subtotal (95% CI)**		**344**		**311**	**68.2%**	**0.79 [0.55, 1.15]**
Total events	42		52			
Heterogeneity: Chi² = 2.84, df = 4 (P = 0.58); I² = 0%						
Test for overall effect: Z = 1.22 (P = 0.22)						
**1.12.2 Bolus**						
Sablok 2015	9	108	11	57	18.7%	0.43 [0.19, 0.98]
Yu 2009	8	60	10	59	13.1%	0.79 [0.33, 1.85]
**Subtotal (95% CI)**		**168**		**116**	**31.8%**	**0.58 [0.32, 1.04]**
Total events	17		21			
Heterogeneity: Chi² = 0.98, df = 1 (P = 0.32); I² = 0%						
Test for overall effect: Z = 1.83 (P = 0.07)						
**Total (95% CI)**		**512**		**427**	**100.0%**	**0.73 [0.53, 0.99]**
Total events	59		73			
Heterogeneity: Chi² = 4.73, df = 6 (P = 0.58); I² = 0%						
Test for overall effect: Z = 2.02 (P = 0.04)						
Test for subgroup differences: Chi² = 0.81, df = 1 (P = 0.37), I² = 0%						

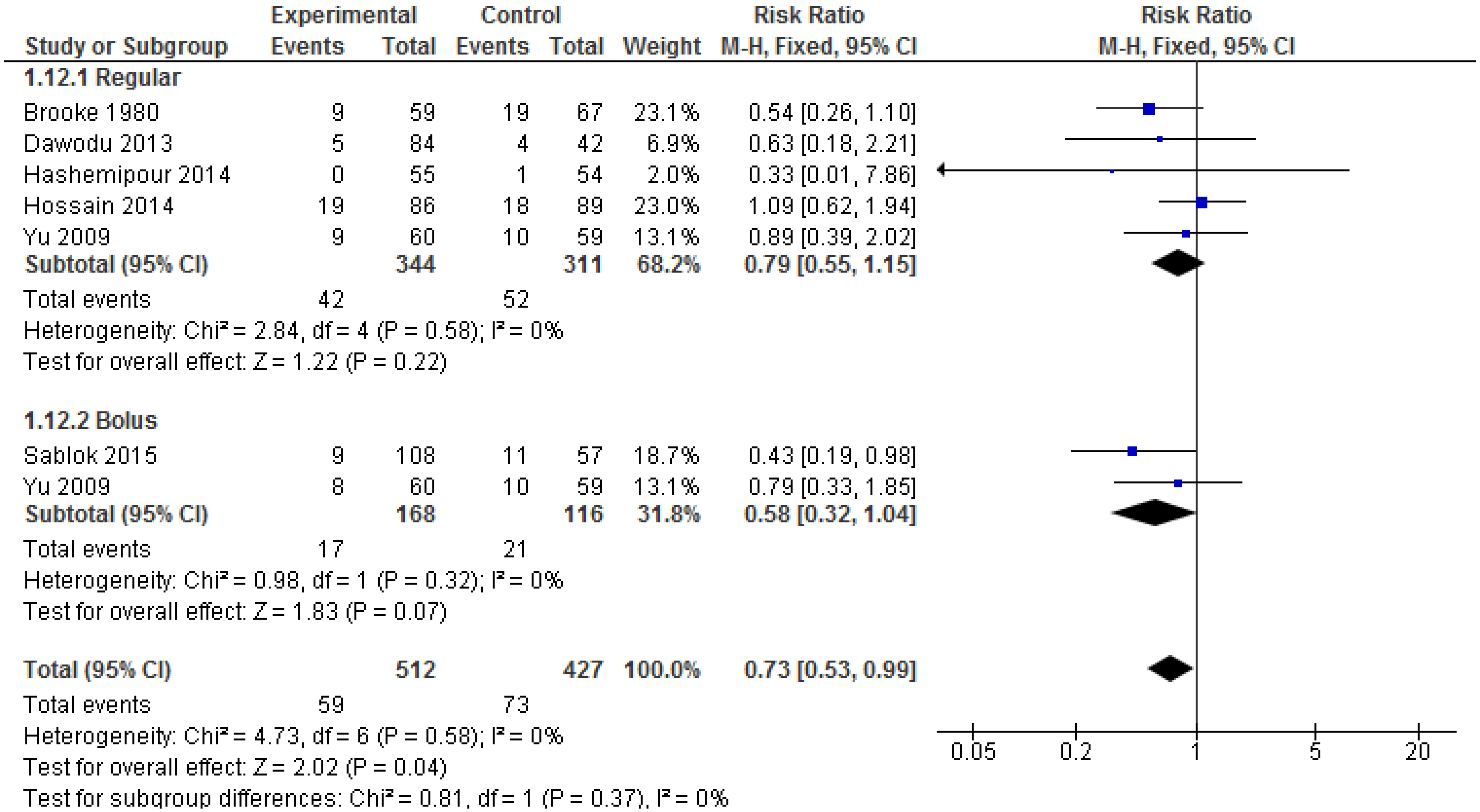

eFigure 2. Summary Risk Ratio of the Association Between Vitamin D Supplementation and Fetal or Neonatal Mortality Stratified by Administration Method of Intervention (Regular or Bolus Dose).

Study or Subgroup	Experimental Events	Experimental Total	Control Events	Control Total	Weight	Risk Ratio M-H, Fixed, 95% CI
**1.20.1 Regular dose**						
Chawes 2016	1	315	3	308	5.5%	0.33 [0.03, 3.12]
Cooper 2016	2	480	7	490	12.4%	0.29 [0.06, 1.40]
Goldring 2013	0	56	3	53	6.5%	0.14 [0.01, 2.56]
Grant 2014	0	167	1	85	3.6%	0.17 [0.01, 4.14]
Hashemipour 2014	0	55	0	54		Not estimable
Hollis 2011	15	239	9	111	22.1%	0.77 [0.35, 1.71]
Hossain 2014	1	86	0	89	0.9%	3.10 [0.13, 75.15]
Litonjua 2016	15	420	14	415	25.3%	1.06 [0.52, 2.17]
Roth 2013	2	73	4	74	7.1%	0.51 [0.10, 2.68]
Yap 2014	2	78	5	84	8.7%	0.43 [0.09, 2.16]
Zerofsky 2016	2	27	1	27	1.8%	2.00 [0.19, 20.77]
**Subtotal (95% CI)**		**1996**		**1790**	**93.8%**	**0.69 [0.45, 1.04]**
Total events	40		47			
Heterogeneity: Chi² = 7.07, df = 9 (P = 0.63); I² = 0%						
Test for overall effect: Z = 1.78 (P = 0.08)						
**1.20.2 Bolus**						
Goldring 2013	0	52	3	53	6.2%	0.15 [0.01, 2.75]
**Subtotal (95% CI)**		**52**		**53**	**6.2%**	**0.15 [0.01, 2.75]**
Total events	0		3			
Heterogeneity: Not applicable						
Test for overall effect: Z = 1.29 (P = 0.20)						
**Total (95% CI)**		**2048**		**1843**	**100.0%**	**0.65 [0.44, 0.98]**
Total events	40		50			
Heterogeneity: Chi² = 8.23, df = 10 (P = 0.61); I² = 0%						
Test for overall effect: Z = 2.05 (P = 0.04)						
Test for subgroup differences: Chi² = 1.05, df = 1 (P = 0.31), I² = 4.8%						

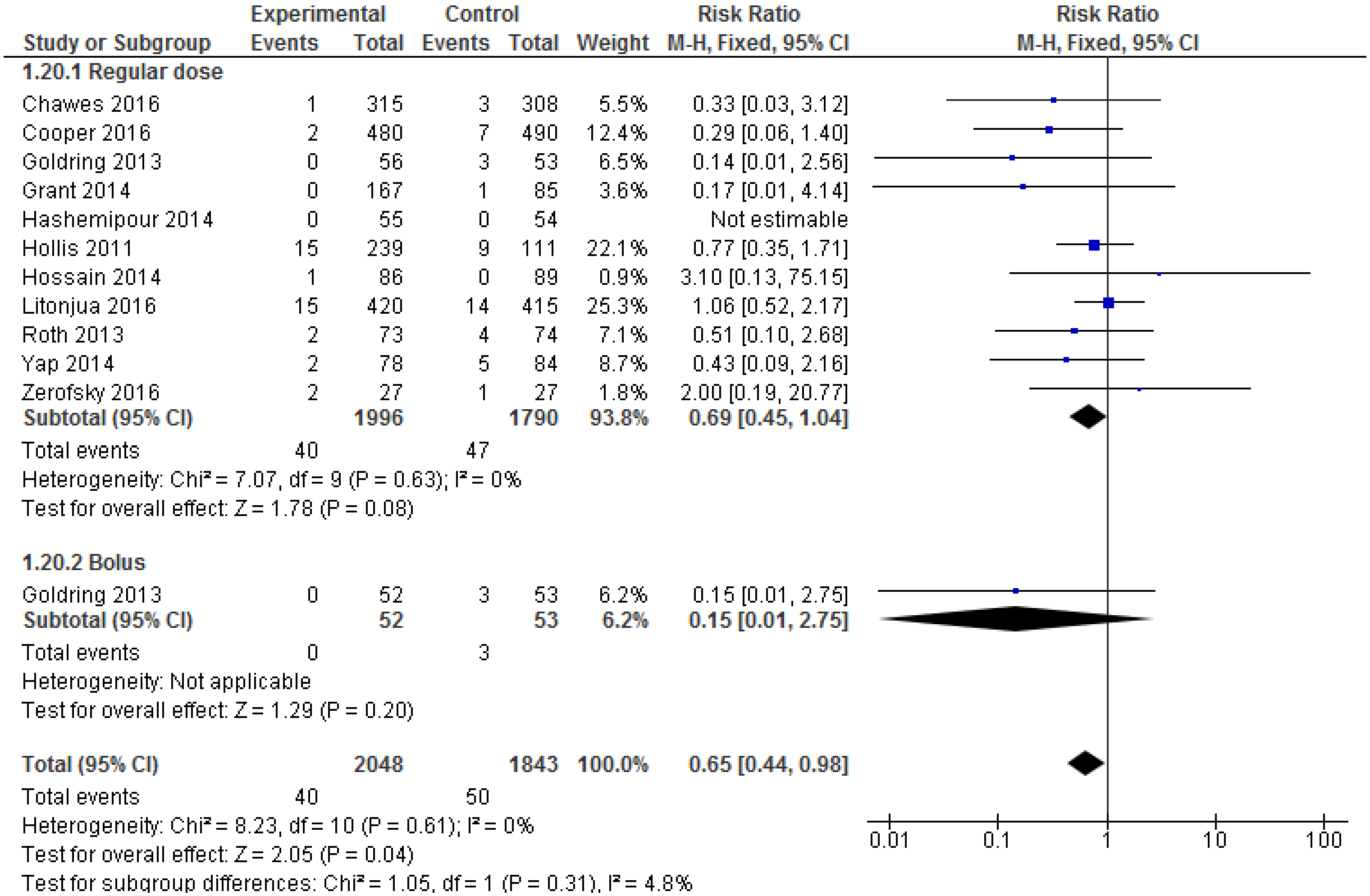

© 2018 BI WG et al. JAMA Pediatrics.. 5

eFigure 3. Forest Plots of Summary Mean Difference (MD) of Neonatal 25-Hydroxyvitamin D (25[OH]D) (ng/mL) Between Vitamin D Supplementation Group and Control Group. Subgroup analyses by (A) Timing (initiation <20 weeks of gestation or ≥20 weeks of gestation); (B) Dose (>2000IU/day or ≤2000IU/day); and (C) Method (regular doses or bolus) of intervention.

(A) Timing

	Experimental			Control				Mean Difference
Study or Subgroup	Mean	SD	Total	Mean	SD	Total	Weight	IV, Random, 95% CI
**1.1.1 Timing of vitamin D supplementation during pregnancy: initiation <20 weeks of gestation**								
Hollis 2011	24.66	10.22	239	18.23	10.14	111	7.6%	6.43 [4.14, 8.72]
Litonjua 2016	28	12.43	303	19.2	9.99	317	7.7%	8.80 [7.02, 10.58]
March 2015	32.77	11.5	65	30.45	10.02	40	7.0%	2.32 [-1.86, 6.50]
Mojibian 2015	37.96	18.03	186	29.75	19.03	203	7.2%	8.21 [4.53, 11.89]
Rodda 2015	32.45	9.49	22	16.83	7.41	23	6.7%	15.62 [10.63, 20.61]
Sahoo 2016	16.72	6.38	36	7.13	5.41	16	7.3%	9.59 [6.22, 12.96]
Yesiltepe Mutlu 2014	27.54	14.75	32	7.53	3.96	19	6.5%	20.01 [14.60, 25.42]
**Subtotal (95% CI)**			**883**			**729**	**49.9%**	**9.69 [6.64, 12.75]**
Heterogeneity: Tau² = 13.42; Chi² = 37.23, df = 6 (P < 0.00001); I² = 84%								
Test for overall effect: Z = 6.22 (P < 0.00001)								
**1.1.2 Timing of vitamin D supplementation during pregnancy: initiation ≥ 20 weeks of gestation**								
Brooke 1980	55.25	33.24	59	4.09	6.56	67	5.2%	51.16 [42.53, 59.79]
Delvin 1986	18.03	2	14	7.01	1	13	7.8%	11.02 [9.84, 12.20]
Goldring 2013	12.21	5.46	68	7.53	3.77	31	7.7%	4.68 [2.82, 6.54]
Hashemipour 2014	27.74	5.21	55	10.92	4.41	54	7.7%	16.82 [15.01, 18.63]
Mallet 1986	6.85	2.1	48	2.12	1	29	7.8%	4.73 [4.03, 5.43]
Roth 2013	41.19	11.46	65	15.63	7.49	67	7.3%	25.56 [22.25, 28.87]
Yap 2014	46.07	19.03	89	29.05	12.02	90	6.8%	17.02 [12.35, 21.69]
**Subtotal (95% CI)**			**398**			**351**	**50.1%**	**17.40 [11.85, 22.94]**
Heterogeneity: Tau² = 52.26; Chi² = 431.53, df = 6 (P < 0.00001); I² = 99%								
Test for overall effect: Z = 6.15 (P < 0.00001)								
**Total (95% CI)**			**1281**			**1080**	**100.0%**	**13.50 [10.12, 16.87]**
Heterogeneity: Tau² = 37.85; Chi² = 469.97, df = 13 (P < 0.00001); I² = 97%								
Test for overall effect: Z = 7.84 (P < 0.00001)								
Test for subgroup differences: Chi² = 5.69, df = 1 (P = 0.02), I² = 82.4%								

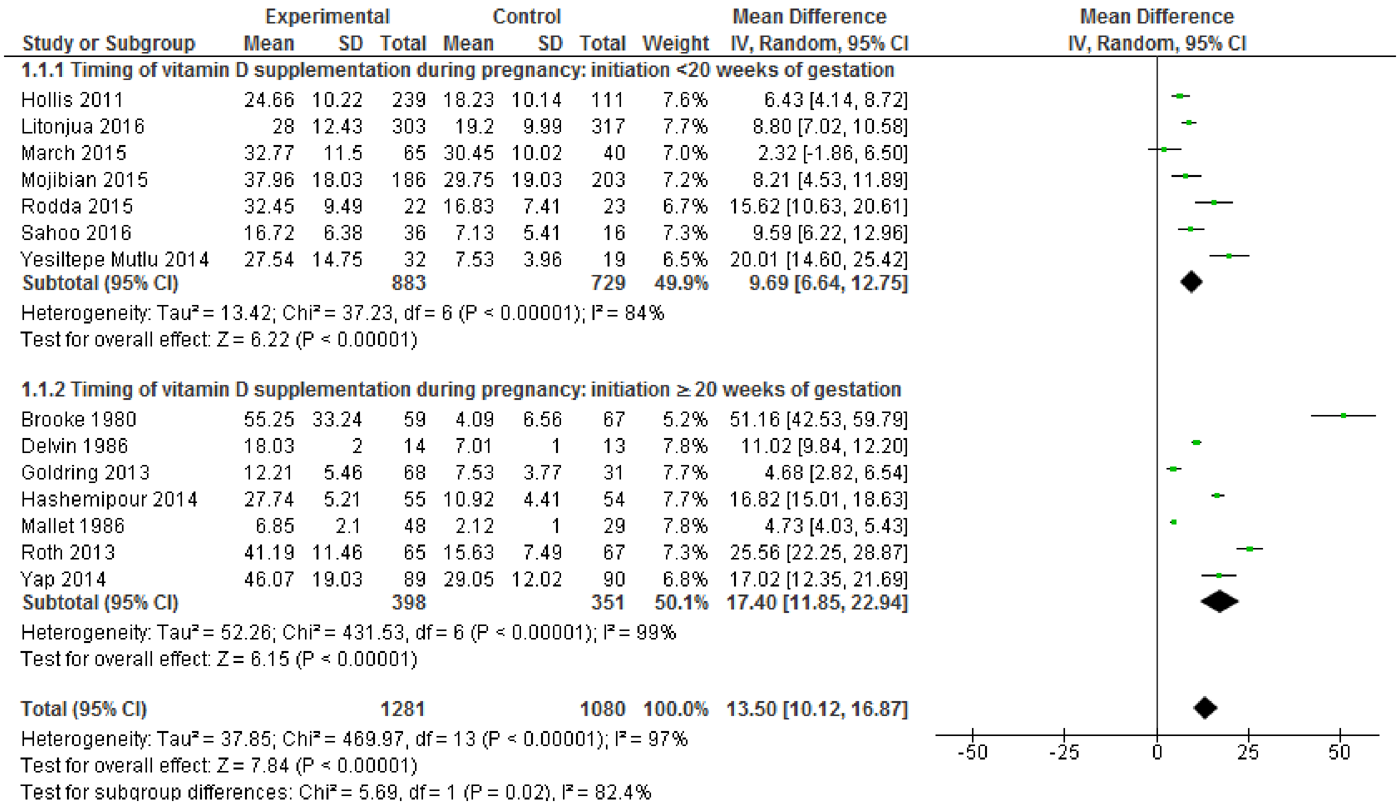

© 2018 BI WG et al. JAMA Pediatrics.. 6

(B) Dose

Study or Subgroup	Experimental Mean	Experimental SD	Experimental Total	Control Mean	Control SD	Control Total	Weight	Mean Difference IV, Random, 95% CI
**1.1.1 Dose > 2000 IU/day**								
Hashemipour 2014	27.74	5.21	55	10.92	4.41	54	6.7%	16.82 [15.01, 18.63]
Hollis 2011	26.56	10.34	117	18.23	10.14	111	6.5%	8.33 [5.67, 10.99]
Litonjua 2016	28	12.43	303	19.2	9.99	317	6.7%	8.80 [7.02, 10.58]
Rodda 2015	32.45	9.49	22	16.83	7.41	23	5.8%	15.62 [10.63, 20.61]
Roth 2013	41.19	11.46	65	15.63	7.49	67	6.4%	25.56 [22.25, 28.87]
Sahoo 2016	19.15	5.53	23	7.13	5.41	16	6.3%	12.02 [8.54, 15.50]
Yap 2014	46.07	19.03	89	29.05	12.02	90	5.9%	17.02 [12.35, 21.69]
**Subtotal (95% CI)**			**674**			**678**	**44.4%**	**14.79 [10.31, 19.27]**
Heterogeneity: Tau² = 33.62; Chi² = 108.68, df = 6 (P < 0.00001); I² = 94%								
Test for overall effect: Z = 6.47 (P < 0.00001)								
**1.1.2 Dose ≤ 2000 IU/day**								
Brooke 1980	55.25	33.24	59	4.09	6.56	67	4.5%	51.16 [42.53, 59.79]
Delvin 1986	18.03	2	14	7.01	1	13	6.8%	11.02 [9.84, 12.20]
Goldring 2013	12.21	5.46	68	7.53	3.77	31	6.7%	4.68 [2.82, 6.54]
Hollis 2011	22.84	9.82	122	18.23	10.14	111	6.6%	4.61 [2.04, 7.18]
Mallet 1986	6.85	2.1	48	2.12	1	29	6.8%	4.73 [4.03, 5.43]
March 2015	32.77	11.5	65	30.45	10.02	40	6.1%	2.32 [-1.86, 6.50]
Mojibian 2015	37.96	18.03	186	29.75	19.03	203	6.3%	8.21 [4.53, 11.89]
Sahoo 2016	12.42	5.61	13	7.13	5.41	16	6.1%	5.29 [1.25, 9.33]
Yesiltepe Mutlu 2014	27.54	14.75	32	7.53	3.97	19	5.7%	20.01 [14.60, 25.42]
**Subtotal (95% CI)**			**607**			**529**	**55.6%**	**10.64 [7.02, 14.26]**
Heterogeneity: Tau² = 26.77; Chi² = 218.69, df = 8 (P < 0.00001); I² = 96%								
Test for overall effect: Z = 5.76 (P < 0.00001)								
**Total (95% CI)**			**1281**			**1207**	**100.0%**	**12.67 [9.58, 15.77]**

Heterogeneity: Tau² = 36.31; Chi² = 480.94, df = 15 (P < 0.00001); I² = 97%
Test for overall effect: Z = 8.02 (P < 0.00001)
Test for subgroup differences: Chi² = 2.00, df = 1 (P = 0.16), I² = 49.9%

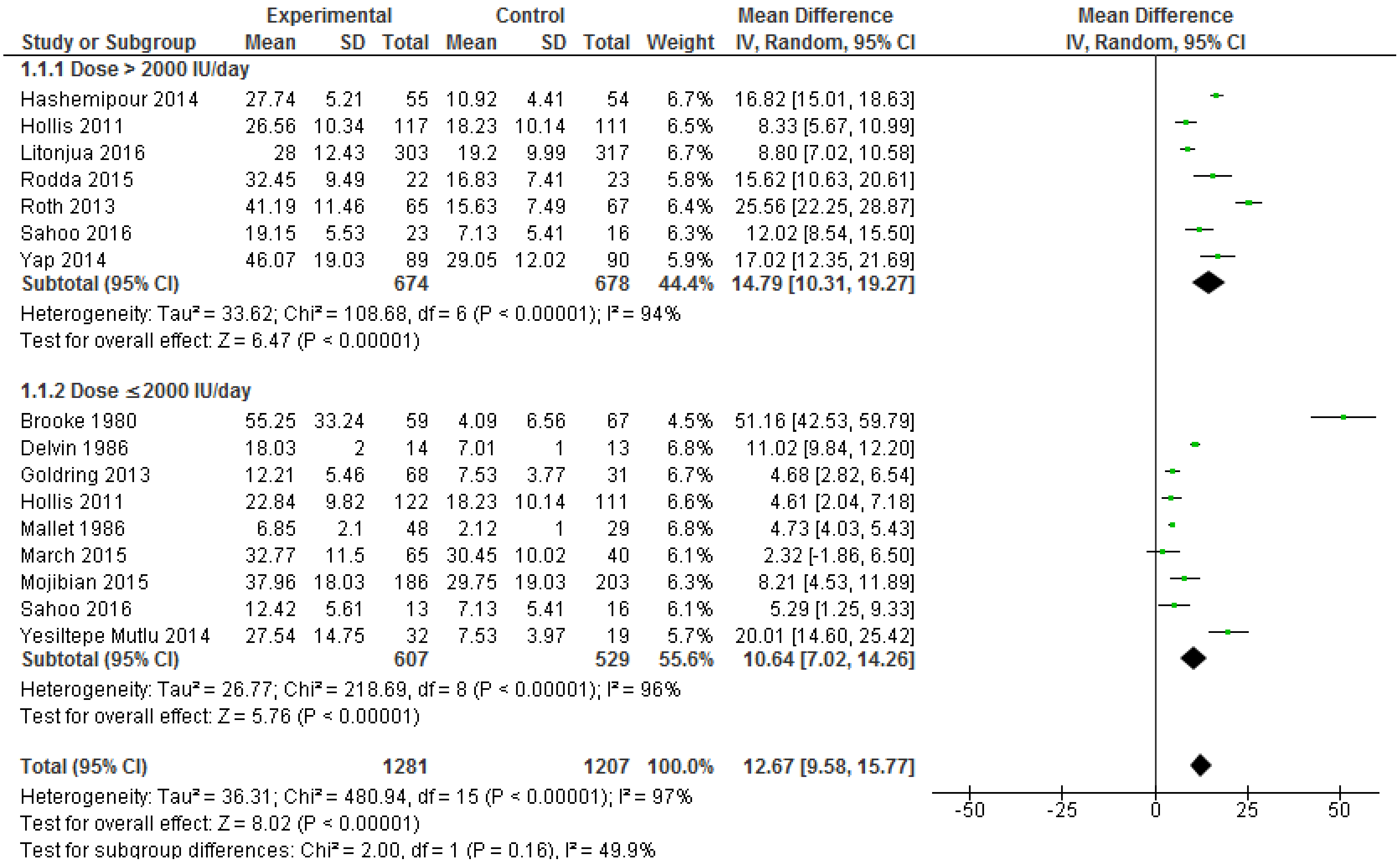

(C) Method (regular doses or bolus)

Study or Subgroup	Experimental Mean	Experimental SD	Experimental Total	Control Mean	Control SD	Control Total	Weight	Mean Difference IV, Random, 95% CI
**1.1.1 Administration method of vitamin D supplementation during pregnancy: Regular doses**								
Brooke 1980	55.25	33.24	59	4.09	6.56	67	4.3%	51.16 [42.53, 59.79]
Delvin 1986	18.03	2	14	7.01	1	13	6.8%	11.02 [9.84, 12.20]
Goldring 2013	13.66	6.53	36	7.53	3.77	31	6.6%	6.13 [3.62, 8.64]
Hashemipour 2014	27.74	5.2	55	10.92	4.41	54	6.7%	16.82 [15.01, 18.63]
Hollis 2011	24.66	10.22	239	18.23	10.14	111	6.6%	6.43 [4.14, 8.72]
Litonjua 2016	28	12.42	303	19.2	9.99	317	6.7%	8.80 [7.02, 10.58]
Mallet 1986	6.29	2.04	21	2.12	1	29	6.8%	4.17 [3.22, 5.12]
March 2015	32.77	11.5	65	30.45	10.02	40	6.0%	2.32 [-1.86, 6.50]
Mojibian 2015	37.96	18.03	186	29.75	19.03	203	6.2%	8.21 [4.53, 11.89]
Rodda 2015	32.45	9.49	22	16.83	7.41	23	5.7%	15.62 [10.63, 20.61]
Roth 2013	41.19	11.46	65	15.63	7.49	67	6.3%	25.56 [22.25, 28.87]
Yap 2014	46.07	19.03	89	29.05	12.02	90	5.8%	17.02 [12.35, 21.69]
Yesiltepe Mutlu 2014	27.54	14.75	32	7.53	3.97	19	5.5%	20.01 [14.60, 25.42]
**Subtotal (95% CI)**			**1186**			**1064**	**80.1%**	**13.97 [10.27, 17.68]**
Heterogeneity: Tau² = 42.70; Chi² = 424.93, df = 12 (P < 0.00001); I² = 97%								
Test for overall effect: Z = 7.39 (P < 0.00001)								
**1.1.2 Administration method of vitamin D supplementation during pregnancy: Bolus**								
Goldring 2013	10.58	3.33	32	7.53	3.77	31	6.7%	3.05 [1.29, 4.81]
Mallet 1986	7.29	2.08	27	2.12	1	29	6.9%	5.17 [4.31, 6.03]
Sahoo 2016	16.72	6.38	36	7.13	5.41	16	6.3%	9.59 [6.22, 12.96]
**Subtotal (95% CI)**			**95**			**76**	**19.9%**	**5.49 [2.96, 8.03]**
Heterogeneity: Tau² = 3.93; Chi² = 11.99, df = 2 (P = 0.002); I² = 83%								
Test for overall effect: Z = 4.25 (P < 0.0001)								
**Total (95% CI)**			**1281**			**1140**	**100.0%**	**12.18 [9.31, 15.05]**
Heterogeneity: Tau² = 31.17; Chi² = 506.89, df = 15 (P < 0.00001); I² = 97%								
Test for overall effect: Z = 8.31 (P < 0.00001)								
Test for subgroup differences: Chi² = 13.70, df = 1 (P = 0.0002), I² = 92.7%								

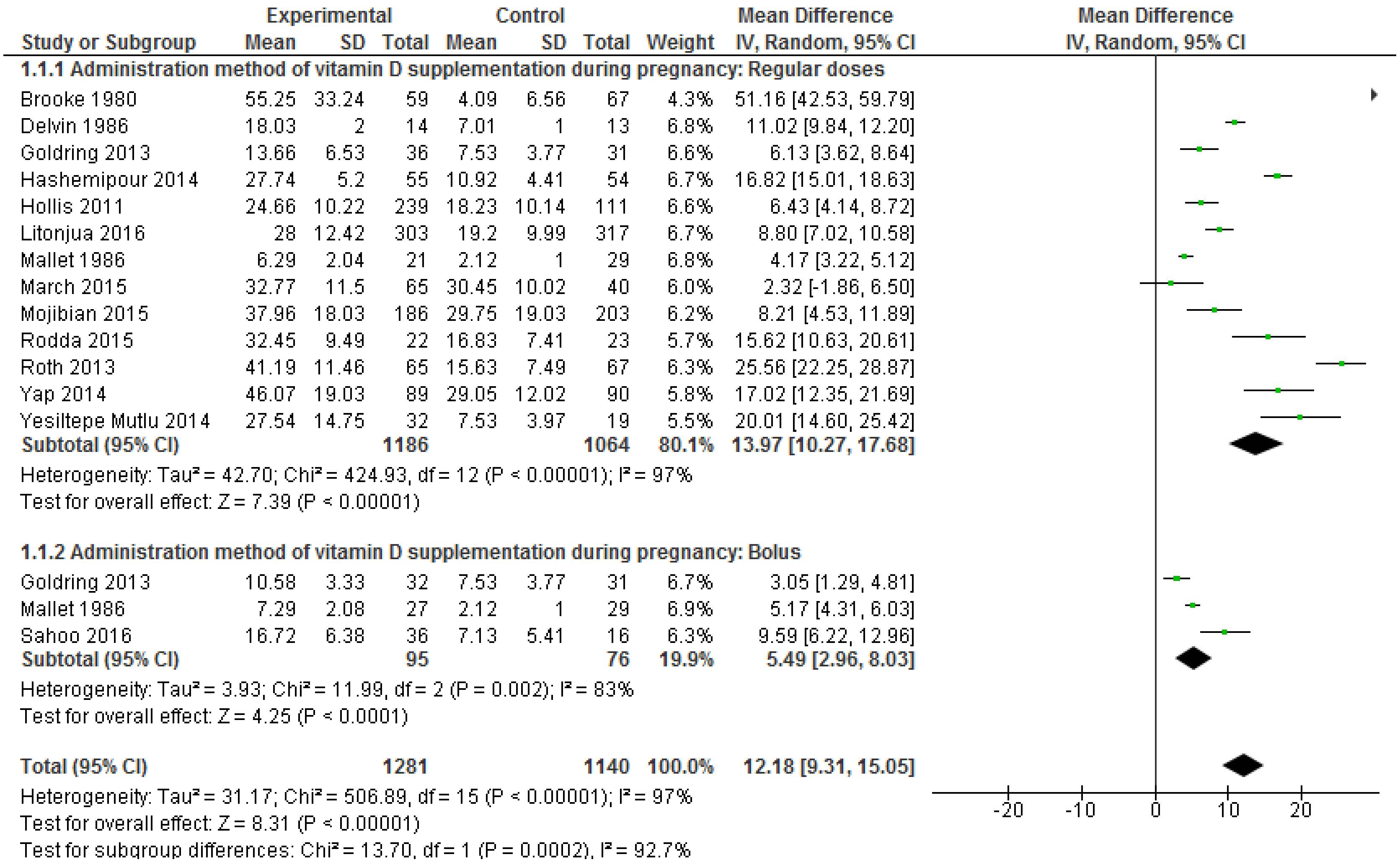

eFigure 4. Forest Plots of Summary Mean Difference (MD) of Neonatal Calcium (mg/dL) Between Vitamin D Supplementation Group and Control Group.

	Experimental			Control				Mean Difference
Study or Subgroup	Mean	SD	Total	Mean	SD	Total	Weight	IV, Random, 95% CI
Brooke 1980	10.84	0.61	59	10.6	0.65	67	13.9%	0.24 [0.02, 0.46]
Delvin 1986	10.2	0.77	15	10.5	0.39	15	8.9%	-0.30 [-0.74, 0.14]
Grant 2014	10.44	0.57	137	10.31	0.53	63	15.3%	0.13 [-0.03, 0.29]
Kalra 2012	8.99	1.6	59	8.8	2	33	4.2%	0.19 [-0.61, 0.99]
Mallet 1986	9.69	0.7	48	9.48	0.44	29	13.1%	0.21 [-0.04, 0.46]
Marya 1988	11.07	0.71	100	10.29	1.03	100	13.3%	0.78 [0.53, 1.03]
Sahoo 2016	9.23	1.14	36	9.2	1.2	16	5.1%	0.03 [-0.67, 0.73]
Yap 2014	10.9	0.7	89	10.7	0.8	90	13.9%	0.20 [-0.02, 0.42]
Yesiltepe Mutlu 2014	8.86	0.48	32	8.9	0.5	19	12.4%	-0.04 [-0.32, 0.24]
**Total (95% CI)**			**575**			**432**	**100.0%**	**0.19 [0.00, 0.38]**

Heterogeneity: $Tau^2 = 0.05$; $Chi^2 = 30.33$, df = 8 (P = 0.0002); $I^2 = 74\%$
Test for overall effect: Z = 1.99 (P = 0.05)

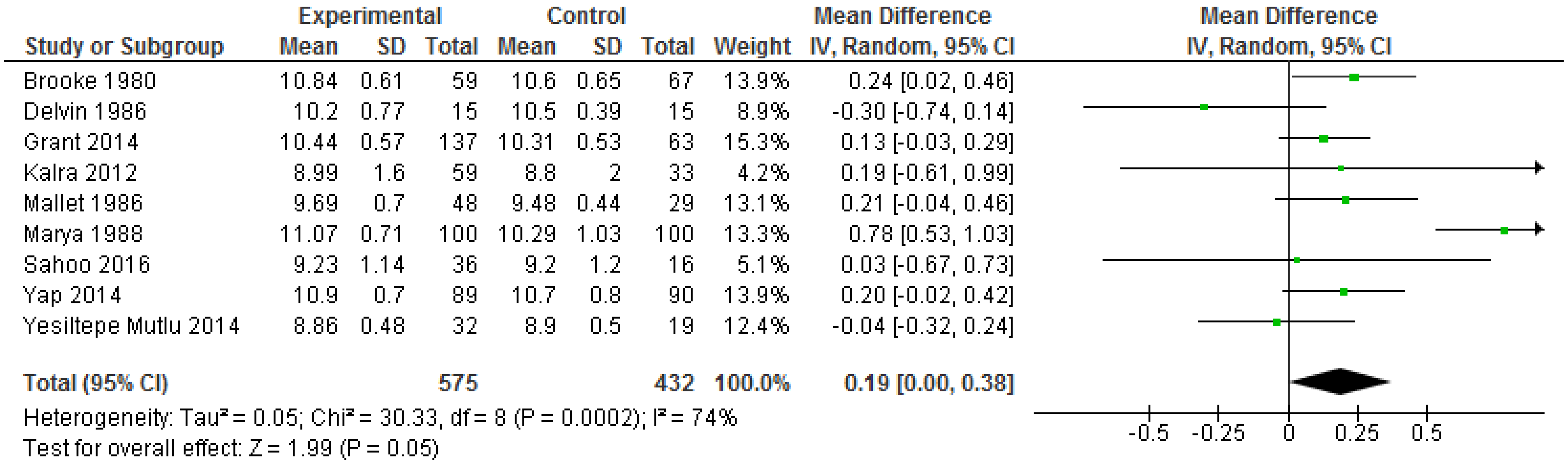

eFigure 5. Forest Plots of Summary Mean Difference (MD) of Birth Weight (g) Between Vitamin D Supplementation Group and Control Group. Subgroup analyses by (A) Timing (initiation <20 weeks of gestation or ≥20 weeks of gestation); (B) Dose (>2000IU/day or ≤2000IU/day); and (C) Method (regular doses or bolus) of intervention.

(A) Timing

Study or Subgroup	Experimental Mean	Experimental SD	Experimental Total	Control Mean	Control SD	Control Total	Weight	Mean Difference IV, Random, 95% CI
**1.3.1 Timing of vitamin D supplementation during pregnancy: initiation <20 weeks of gestation**								
Cooper 2016	3,481	543	479	3,518	517	486	9.5%	-37.00 [-103.91, 29.91]
Hollis 2011	3,323.14	591.17	239	3,221.8	674.9	111	6.1%	101.34 [-44.88, 247.56]
Kalra 2012	3,055.35	3,929.62	71	2,770	3,990	38	0.1%	285.35 [-1278.26, 1848.96]
Litonjua 2016	3,267.9	543.2	402	3,282.5	588.5	401	9.0%	-14.60 [-92.94, 63.74]
Mojibian 2015	3,088.72	481.21	186	3,125.57	434.9	203	8.4%	-36.85 [-128.29, 54.59]
Sahoo 2016	2,710	450	36	2,670	430	16	3.2%	40.00 [-216.91, 296.91]
Yesiltepe Mutlu 2014	3,367.5	424.5	32	3,375	546	19	2.7%	-7.50 [-293.69, 278.69]
Zerofsky 2016	3,668	431	24	3,379	557	25	2.8%	289.00 [10.78, 567.22]
**Subtotal (95% CI)**			**1469**			**1299**	**41.9%**	**-5.21 [-52.30, 41.88]**
Heterogeneity: Tau² = 547.65; Chi² = 7.92, df = 7 (P = 0.34); I² = 12%								
Test for overall effect: Z = 0.22 (P = 0.83)								
**1.3.2 Timing of vitamin D supplementation during pregnancy: initiation ≥ 20 weeks of gestation**								
Brooke 1980	3,157	468.5	59	3,034	523.9	67	5.2%	123.00 [-50.29, 296.29]
Goldring 2013	3,307	497	108	3,268	585	50	4.8%	39.00 [-148.29, 226.29]
Hashemipour 2014	3,429	351.9	55	3,258.8	328.2	54	6.9%	170.20 [42.48, 297.92]
Hossain 2014	2,810	520	86	2,750	440	89	6.3%	60.00 [-82.95, 202.95]
Karamali 2015	3,313.6	341.1	30	3,141	495.9	30	4.0%	172.60 [-42.78, 387.98]
Mallet 1986	3,280	429.7	48	3,460	376.9	29	4.9%	-180.00 [-363.29, 3.29]
Marya 1988	2,990	360	100	2,800	370	100	8.0%	190.00 [88.82, 291.18]
Roth 2013	2,802	543	73	2,788	378	74	5.9%	14.00 [-137.44, 165.44]
Sablok 2015	2,600	410	108	2,400	310	57	7.5%	200.00 [88.39, 311.61]
Yap 2014	3,337	671	89	3,267	649	90	4.6%	70.00 [-123.42, 263.42]
**Subtotal (95% CI)**			**756**			**640**	**58.1%**	**97.74 [29.40, 166.08]**
Heterogeneity: Tau² = 5940.87; Chi² = 18.41, df = 9 (P = 0.03); I² = 51%								
Test for overall effect: Z = 2.80 (P = 0.005)								
**Total (95% CI)**			**2225**			**1939**	**100.0%**	**64.03 [9.95, 118.11]**
Heterogeneity: Tau² = 6842.07; Chi² = 41.99, df = 17 (P = 0.0007); I² = 60%								
Test for overall effect: Z = 2.32 (P = 0.02)								
Test for subgroup differences: Chi² = 5.91, df = 1 (P = 0.02), I² = 83.1%								

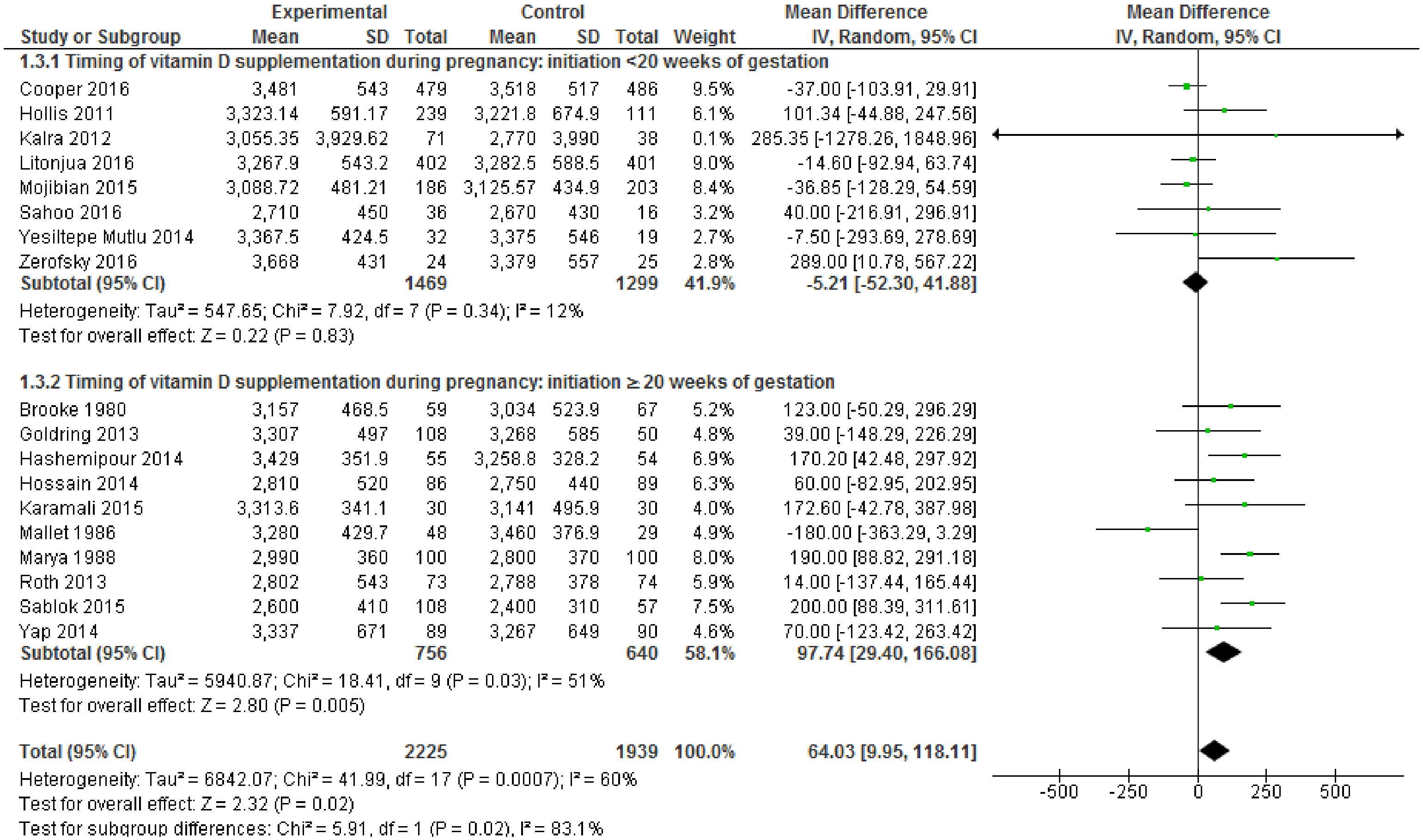

(B) Dose

Study or Subgroup	Experimental Mean	Experimental SD	Experimental Total	Control Mean	Control SD	Control Total	Weight	Mean Difference IV, Random, 95% CI
**1.3.1 Dose > 2000 IU/day**								
Hashemipour 2014	3,429	351.9	55	3,258.8	328.2	54	6.8%	170.20 [42.48, 297.92]
Hollis 2011	3,284.6	597.6	117	3,221.8	674.9	111	5.2%	62.80 [-103.00, 228.60]
Hossain 2014	2,810	520	86	2,750	440	89	6.1%	60.00 [-82.95, 202.95]
Karamali 2015	3,313.6	341.1	30	3,141	495.9	30	3.7%	172.60 [-42.78, 387.98]
Litonjua 2016	3,267.9	543.2	402	3,282.5	588.5	401	9.5%	-14.60 [-92.94, 63.74]
Marya 1988	2,990	360	100	2,800	370	100	8.2%	190.00 [88.82, 291.18]
Mojibian 2015	3,088.72	481.21	186	3,125.57	434.9	203	8.7%	-36.85 [-128.29, 54.59]
Roth 2013	2,802	543	73	2,788	378	74	5.7%	14.00 [-137.44, 165.44]
Sablok 2015	2,600	410	108	2,400	310	57	7.6%	200.00 [88.39, 311.61]
Sahoo 2016	2,670	450	23	2,670	430	16	2.5%	0.00 [-279.67, 279.67]
Yap 2014	3,337	671	89	3,267	649	90	4.3%	70.00 [-123.42, 263.42]
**Subtotal (95% CI)**			**1269**			**1225**	**68.2%**	**80.64 [17.83, 143.44]**
Heterogeneity: Tau² = 6063.36; Chi² = 24.37, df = 10 (P = 0.007); I² = 59%								
Test for overall effect: Z = 2.52 (P = 0.01)								
**1.3.2 Dose ≤ 2000 IU/day**								
Brooke 1980	3,157	468.5	59	3,034	523.9	67	4.9%	123.00 [-50.29, 296.29]
Cooper 2016	3,481	543	479	3,518	517	486	10.1%	-37.00 [-103.91, 29.91]
Goldring 2013	3,307	497	108	3,268	585	50	4.5%	39.00 [-148.29, 226.29]
Hollis 2011	3,360.1	585	122	3,221.8	674.9	111	5.3%	138.30 [-24.61, 301.21]
Kalra 2012	3,055.35	3,929.62	71	2,770	3,990	38	0.1%	285.35 [-1278.26, 1848.96]
Sahoo 2016	2,780	460	13	2,670	430	16	1.9%	110.00 [-216.99, 436.99]
Yesiltepe Mutlu 2014	3,367.5	424.5	32	3,375	546	19	2.4%	-7.50 [-293.69, 278.69]
Zerofsky 2016	3,668	431	24	3,379	557	25	2.5%	289.00 [10.78, 567.22]
**Subtotal (95% CI)**			**908**			**812**	**31.8%**	**61.79 [-21.30, 144.88]**
Heterogeneity: Tau² = 4055.86; Chi² = 10.24, df = 7 (P = 0.18); I² = 32%								
Test for overall effect: Z = 1.46 (P = 0.14)								
**Total (95% CI)**			**2177**			**2037**	**100.0%**	**75.71 [26.19, 125.23]**
Heterogeneity: Tau² = 5153.27; Chi² = 36.93, df = 18 (P = 0.005); I² = 51%								
Test for overall effect: Z = 3.00 (P = 0.003)								
Test for subgroup differences: Chi² = 0.13, df = 1 (P = 0.72), I² = 0%								

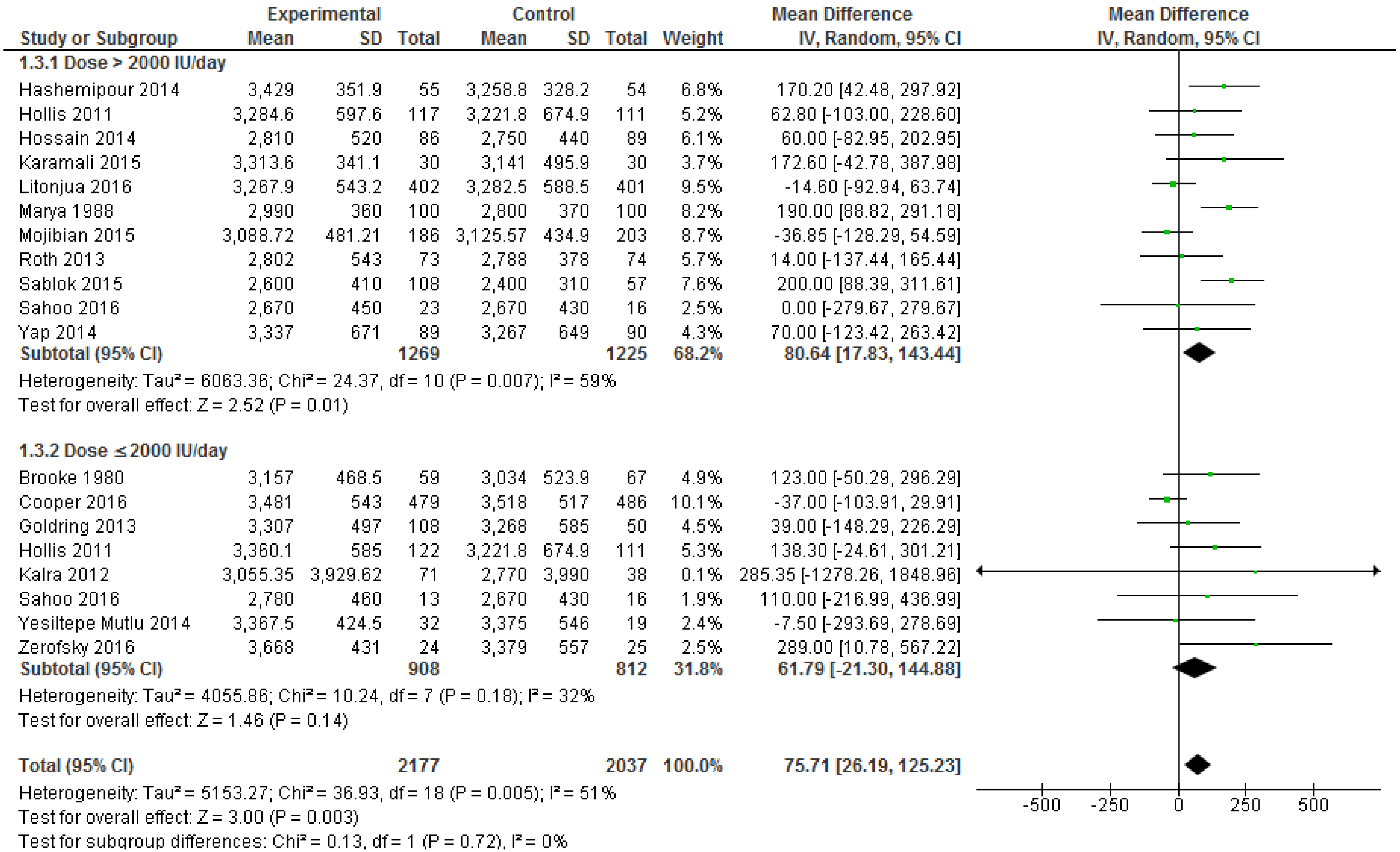

© 2018 BI WG et al. JAMA Pediatrics.. 11

(C) Method (regular doses or bolus)

Study or Subgroup	Experimental Mean	Experimental SD	Experimental Total	Control Mean	Control SD	Control Total	Weight	Mean Difference IV, Random, 95% CI
**1.3.1 Administration method of vitamin D supplementation during pregnancy: Regular doses**								
Brooke 1980	3,157	468.5	59	3,034	523.9	67	5.4%	123.00 [-50.29, 296.29]
Cooper 2016	3,481	543	479	3,518	517	486	10.5%	-37.00 [-103.91, 29.91]
Hashemipour 2014	3,429	351.9	55	3,258.8	328.2	54	7.3%	170.20 [42.48, 297.92]
Hollis 2011	3,323.14	591.17	239	3,221.8	674.9	111	6.4%	101.34 [-44.88, 247.56]
Hossain 2014	2,810	520	86	2,750	440	89	6.6%	60.00 [-82.95, 202.95]
Kalra 2012	3,055.35	3,929.62	71	2,770	3,990	38	0.1%	285.35 [-1278.26, 1848.96]
Karamal 2015	3,313.6	341.1	30	3,141	495.9	30	4.1%	172.60 [-42.78, 387.98]
Litonjua 2016	3,267.9	543.2	402	3,282.5	588.5	401	9.9%	-14.60 [-92.94, 63.74]
Marya 1988	2,990	360	100	2,800	370	100	8.6%	190.00 [88.82, 291.18]
Mojibian 2015	3,088.72	481.21	186	3,125.57	434.9	203	9.2%	-36.85 [-128.29, 54.59]
Roth 2013	2,802	543	73	2,788	378	74	6.2%	14.00 [-137.44, 165.44]
Sablok 2015	2,600	410	108	2,400	310	57	8.1%	200.00 [88.39, 311.61]
Yap 2014	3,337	671	89	3,267	649	90	4.7%	70.00 [-123.42, 263.42]
Yesiltepe Mutlu 2014	3,367.5	424.5	32	3,375	546	19	2.7%	-7.50 [-293.69, 278.69]
Zerofsky 2016	3,668	431	24	3,379	557	25	2.8%	289.00 [10.78, 567.22]
**Subtotal (95% CI)**			**2033**			**1844**	**92.5%**	**79.64 [22.43, 136.86]**
Heterogeneity: Tau² = 6576.25; Chi² = 36.00, df = 14 (P = 0.001); I² = 61%								
Test for overall effect: Z = 2.73 (P = 0.006)								
**1.3.2 Administration method of vitamin D supplementation during pregnancy: Bolus**								
Goldring 2013	3,290	467	52	3,268	585	50	4.3%	22.00 [-183.92, 227.92]
Sahoo 2016	2,780	450	36	2,670	430	16	3.2%	110.00 [-146.91, 366.91]
**Subtotal (95% CI)**			**88**			**66**	**7.5%**	**56.42 [-104.25, 217.10]**
Heterogeneity: Tau² = 0.00; Chi² = 0.27, df = 1 (P = 0.60); I² = 0%								
Test for overall effect: Z = 0.69 (P = 0.49)								
**Total (95% CI)**			**2121**			**1910**	**100.0%**	**77.19 [24.27, 130.11]**
Heterogeneity: Tau² = 5779.27; Chi² = 36.28, df = 16 (P = 0.003); I² = 56%								
Test for overall effect: Z = 2.86 (P = 0.004)								
Test for subgroup differences: Chi² = 0.07, df = 1 (P = 0.79), I² = 0%								

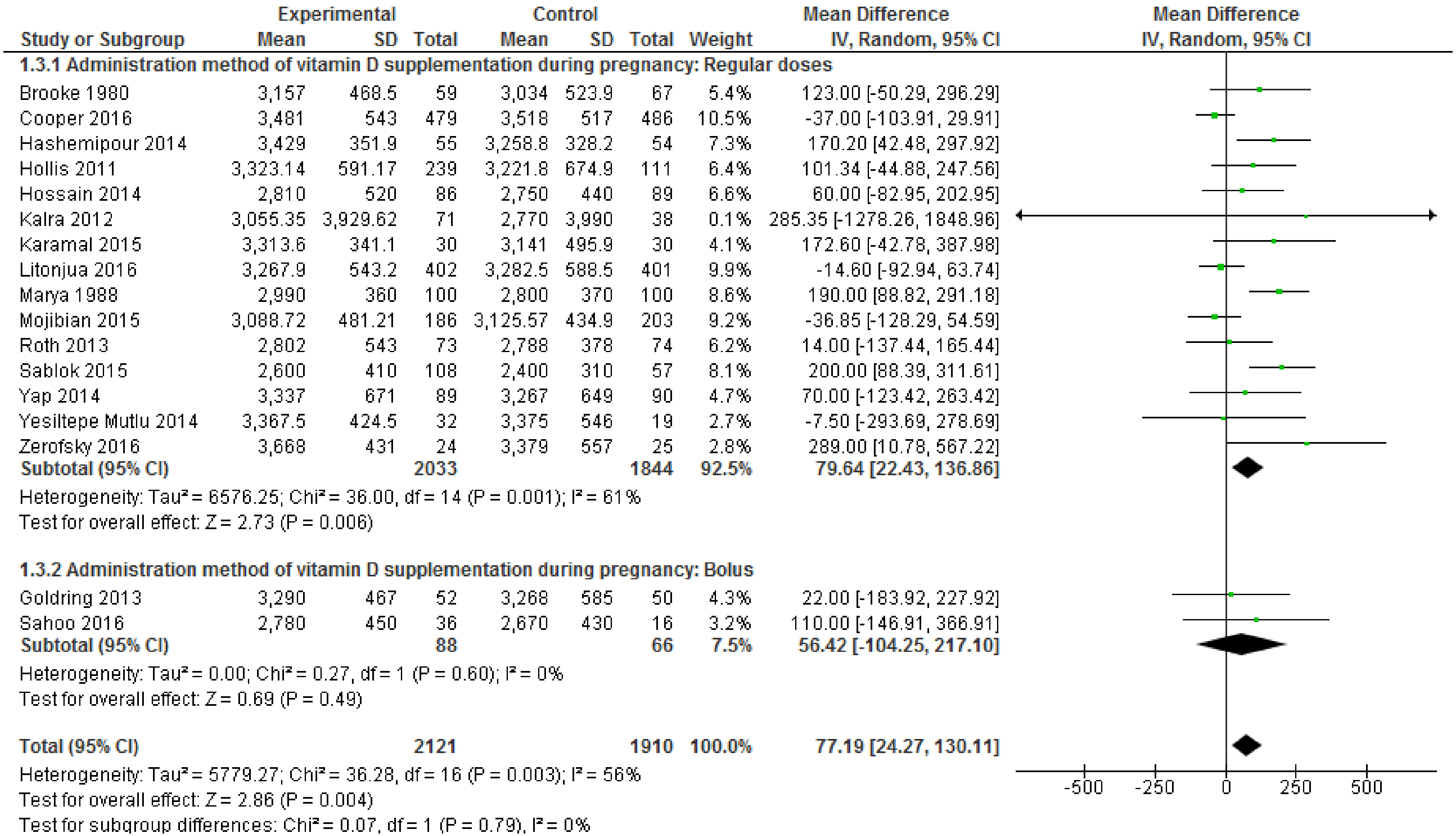

eFigure 6. Forest Plots of Summary Mean Difference (MD) of Postnatal Weight (kg) Between Vitamin D Supplementation Group and Control Group in Infants at Age (A) 3 Months; (B) 6 Months; (C) 9 Months; and (D) 12 Months.

(A)

	Experimental			Control				Mean Difference	Mean Difference
Study or Subgroup	Mean	SD	Total	Mean	SD	Total	Weight	IV, Fixed, 95% CI	IV, Fixed, 95% CI
Brooke 1981	5.65	0.58	53	5.4	0.54	64	14.0%	0.25 [0.05, 0.45]	
Kalra 2012	5.9	0.2	64	5.7	0.2	35	86.0%	0.20 [0.12, 0.28]	
**Total (95% CI)**			**117**			**99**	**100.0%**	**0.21 [0.13, 0.28]**	

Heterogeneity: Chi² = 0.20, df = 1 (P = 0.66); I² = 0%
Test for overall effect: Z = 5.31 (P < 0.00001)

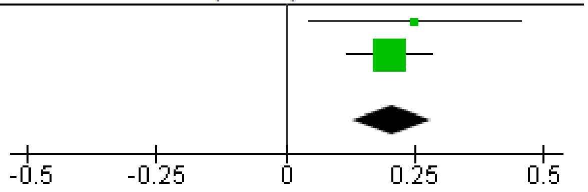

(B)

	Experimental			Control				Mean Difference	Mean Difference
Study or Subgroup	Mean	SD	Total	Mean	SD	Total	Weight	IV, Fixed, 95% CI	IV, Fixed, 95% CI
Brooke 1981	7.56	0.6	53	7.09	0.59	64	33.2%	0.47 [0.25, 0.69]	
Kalra 2012	7.25	0.4	52	6.8	0.3	30	66.8%	0.45 [0.30, 0.60]	
**Total (95% CI)**			**105**			**94**	**100.0%**	**0.46 [0.33, 0.58]**	

Heterogeneity: Chi² = 0.02, df = 1 (P = 0.88); I² = 0%
Test for overall effect: Z = 7.17 (P < 0.00001)

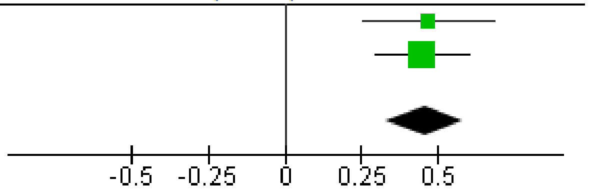

(C)

Study or Subgroup	Experimental Mean	Experimental SD	Experimental Total	Control Mean	Control SD	Control Total	Weight	Mean Difference IV, Random, 95% CI
Brooke 1981	8.54	0.59	53	8.29	0.57	64	50.6%	0.25 [0.04, 0.46]
Kalra 2012	8.45	0.55	40	7.7	0.4	22	49.4%	0.75 [0.51, 0.99]
**Total (95% CI)**			**93**			**86**	**100.0%**	**0.50 [0.01, 0.99]**

Heterogeneity: Tau² = 0.11; Chi² = 9.44, df = 1 (P = 0.002); I² = 89%
Test for overall effect: Z = 1.99 (P = 0.05)

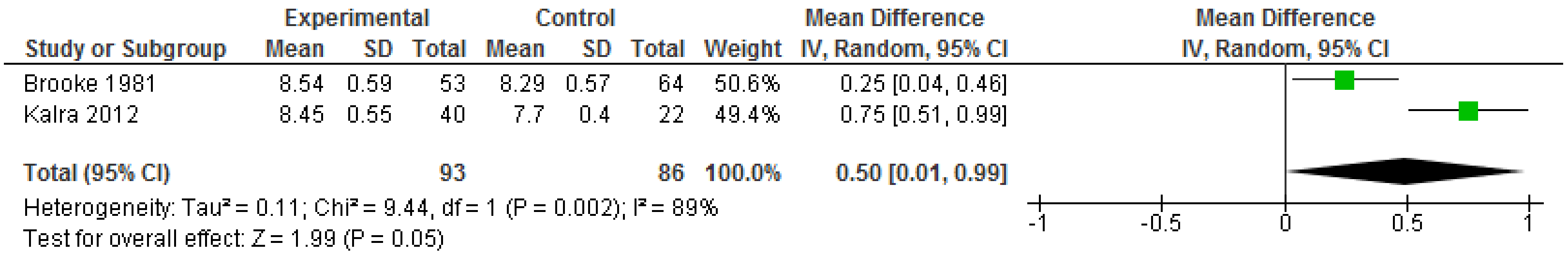

(D)

Study or Subgroup	Experimental Mean	Experimental SD	Experimental Total	Control Mean	Control SD	Control Total	Weight	Mean Difference IV, Fixed, 95% CI
Brooke 1981	9.39	0.66	53	8.98	0.62	64	71.9%	0.41 [0.18, 0.64]
Roth 2013	8.5	1.2	66	8.4	1	69	28.1%	0.10 [-0.27, 0.47]
**Total (95% CI)**			**119**			**133**	**100.0%**	**0.32 [0.12, 0.52]**

Heterogeneity: Chi² = 1.90, df = 1 (P = 0.17); I² = 47%
Test for overall effect: Z = 3.19 (P = 0.001)

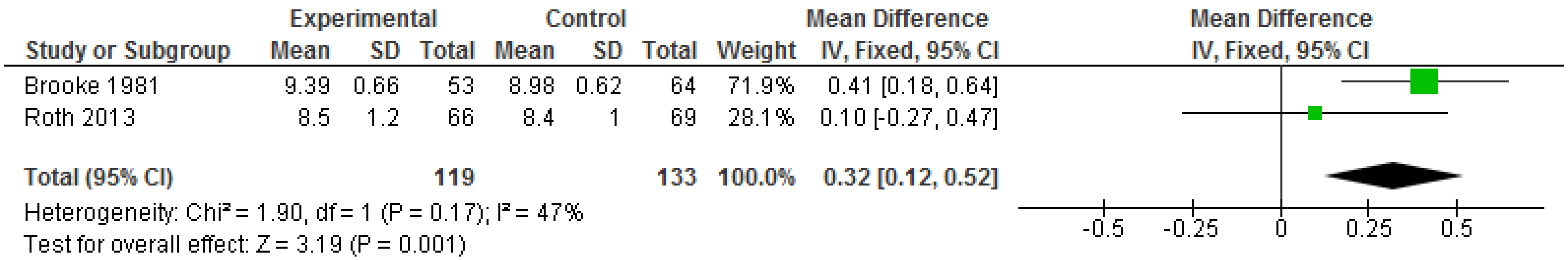

eFigure 7. The Funnel Plots of the Primary Outcomes. (A) Funnel plots for SGA; (B) Funnel plots for fetal or neonatal mortality.

(A) Funnel plots for SGA

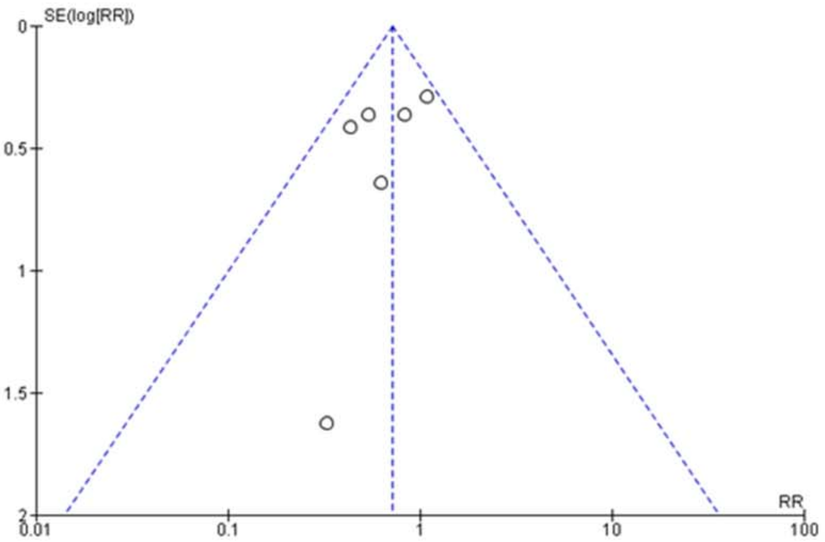

(B) Funnel plots for fetal or neonatal mortality

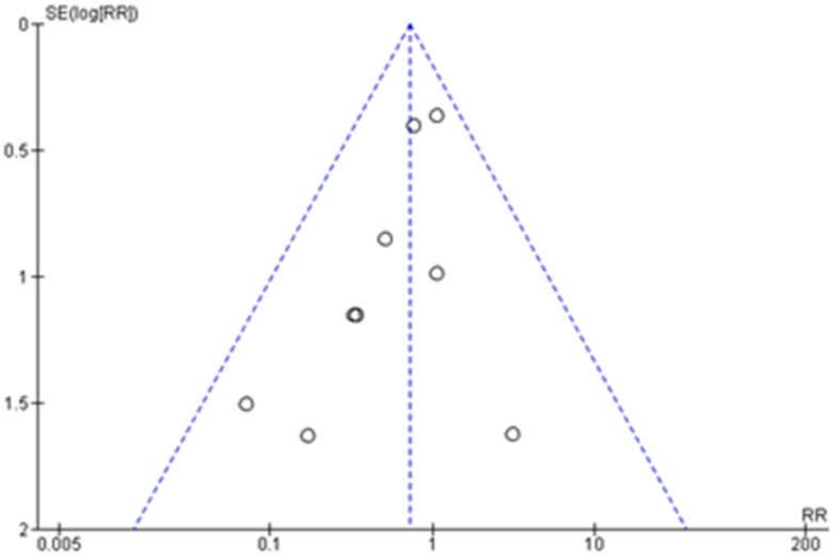

Supplement: Supplement. — eTable. Assessment of Bias Risk of Randomized Clinical Trials eFigure 1. Summary Crude Risk Ratio (RR) of the Association Between Vitamin D Supplementation and Small for Gestational Age (SGA) Stratified by Administration Method of Intervention (Regular or Bolus Dose) eFigure 2. Summary Risk Ratio of the Association 1 Between Vitamin D Supplementation Group and Fetal or Neonatal Mortality Stratified by Administration Method of Intervention (Regular or Bolus Dose). eFigure 3. Forest Plots of Summary Mean Difference (MD) of Neonatal 25-Hydroxyvitamin D (25[OH]D) (ng/mL) Between Vitamin D Supplementation Group and Control Group eFigure 4. Forest Plots of Summary Mean Difference (MD) of Neonatal Calcium (mg/dL) Between Vitamin D Supplementation Group and Control Group eFigure 5. Forest Plots of Summary Mean Difference (MD) of Birth Weight (g) Between Vitamin D Supplementation Group and Control Group eFigure 6. Forest Plots of Summary Mean Difference (MD) of Postnatal Weight (kg) Between Vitamin D Supplementation Group and Control Group in Infants at Age 3 Months; 6 Months; 9 Months; and 12 Months eFigure 7. The Funnel Plots of the Primary Outcomes [file jamapediatr-172-635-s001.pdf]
